# Interleukin-4 receptor signaling modulates neuronal network activity

**DOI:** 10.1084/jem.20211887

**Published:** 2022-05-19

**Authors:** Nicholas Hanuscheck, Carine Thalman, Micaela Domingues, Samantha Schmaul, Muthuraman Muthuraman, Florian Hetsch, Manuela Ecker, Heiko Endle, Mohammadsaleh Oshaghi, Gianvito Martino, Tanja Kuhlmann, Katarzyna Bozek, Tim van Beers, Stefan Bittner, Jakob von Engelhardt, Johannes Vogt, Christina Francisca Vogelaar, Frauke Zipp

**Affiliations:** 1 Department of Neurology, Focus Program Translational Neuroscience and Immunotherapy, Rhine Main Neuroscience Network (rmn2), University Medical Center of the Johannes Gutenberg University Mainz, Mainz, Germany; 2 Institute for Pathophysiology, Focus Program Translational Neuroscience, University Medical Center of the Johannes Gutenberg University Mainz, Mainz, Germany; 3 Department of Molecular and Translational Neuroscience, Cluster of Excellence-Cellular Stress Response in Aging-Associated Diseases and Center of Molecular Medicine Cologne, University of Cologne, Faculty of Medicine and University Hospital Cologne, Cologne, Germany; 4 Neuroimmunology Unit, Institute of Experimental Neurology, Division of Neuroscience, IRCCS San Raffaele Scientific Institute and Vita Salute San Raffaele University, Milan, Italy; 5 Institute for Neuropathology, University Hospital Münster, Münster, Germany; 6 Center for Molecular Medicine, Faculty of Medicine and University Hospital Cologne; University of Cologne, Cologne, Germany; 7 Molecular Cell Biology, Institute I of Anatomy, University of Cologne, Cologne, Germany

## Abstract

Evidence is emerging that immune responses not only play a part in the central nervous system (CNS) in diseases but may also be relevant for healthy conditions. We discovered a major role for the interleukin-4 (IL-4)/IL-4 receptor alpha (IL-4Rα) signaling pathway in synaptic processes, as indicated by transcriptome analysis in IL-4Rα–deficient mice and human neurons with/without IL-4 treatment. Moreover, IL-4Rα is expressed presynaptically, and locally available IL-4 regulates synaptic transmission. We found reduced synaptic vesicle pools, altered postsynaptic currents, and a higher excitatory drive in cortical networks of IL-4Rα–deficient neurons. Acute effects of IL-4 treatment on postsynaptic currents in wild-type neurons were mediated via PKCγ signaling release and led to increased inhibitory activity supporting the findings in IL-4Rα–deficient neurons. In fact, the deficiency of IL-4Rα resulted in increased network activity in vivo, accompanied by altered exploration and anxiety-related learning behavior; general learning and memory was unchanged. In conclusion, neuronal IL-4Rα and its presynaptic prevalence appear relevant for maintaining homeostasis of CNS synaptic function.

## Teaser

Neuroimmune interaction influences essential CNS processes. Hanuscheck et al. report on the expression of interleukin-4 receptor alpha at presynaptic terminals in mouse and the human brain. The modulation of neuronal IL-4R signaling alters the neuronal transcriptome, impacts synaptic transmission, and causes anxiety-related behavior.

## Graphical Abstract

**Figure:**
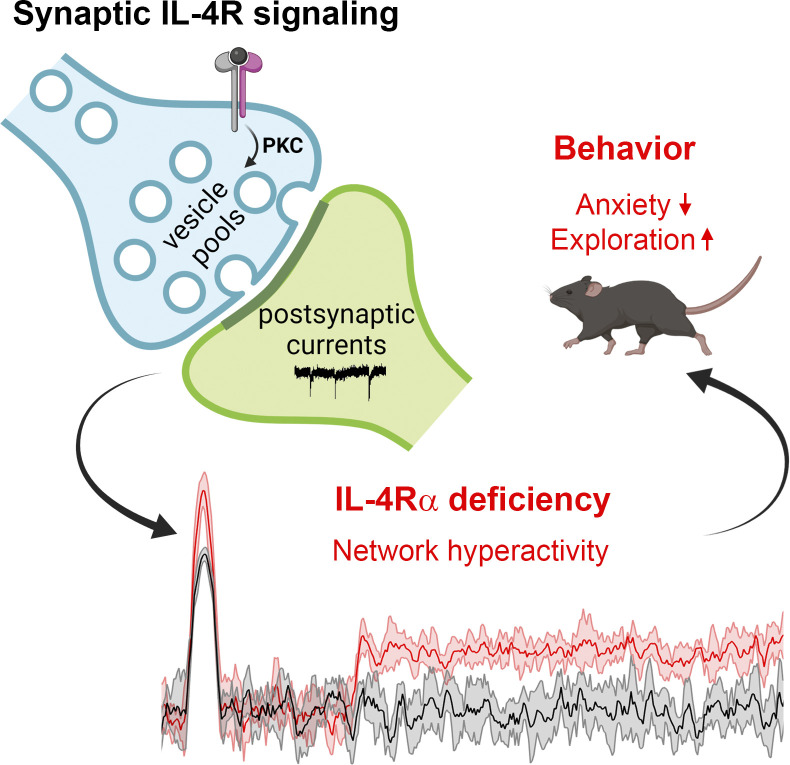

## Introduction

To date, the influence of cytokines on neurons is mainly classified as detrimental. During neuroinflammation, immune cells, among them T (helper) lymphocytes (T cells, e.g., T_H_1 and T_H_2), infiltrate the central nervous system (CNS) and attack resident cells, causing demyelination and degeneration of axons leading to progressive disability (Liblau et al., 2013). Proinflammatory cytokines such as IL-17 (Alves de Lima et al., 2020; Luchtman et al., 2014), TNFα, and IL-1β (Allan et al., 2005; Rizzo et al., 2018; Walsh et al., 2014) were reported to be involved in anxiety, neuronal dysfunction, or even damage in different neurodegenerative and neuroinflammatory diseases (Glass et al., 2010; Liblau et al., 2013). At the same time, regulatory T cells (Treg), isolated from patients with neurodegenerative diseases, display compromised immunomodulatory functions (Faridar et al., 2020; Thome et al., 2021).

Even under healthy conditions, T cells are capable of populating the CNS borders, e.g., skull, meninges, choroid plexus, and perivascular space, and are involved in surveillance and communication of the CNS with the periphery (Filiano et al., 2015; Louveau et al., 2015; Siffrin et al., 2009). Ablation of meningeal lymphatics in homeostasis leads to cognitive impairment (Da Mesquita et al., 2018). Early observations revealed poor cognitive performance in mice deficient of T cells, and the passive transfer of T cells improved cognition (Kipnis et al., 2004; Ziv et al., 2006). Neutralization of corticosteroid signaling in the choroid plexus attenuated anxiety-like behavior under severe psychological stress via the CNS recruitment of T_H_2 and Treg cells (Kertser et al., 2019).

In line with this, it was reported that immune cells and their cytokines can also play beneficial roles in CNS repair (Ellwardt et al., 2016; Popovich, 2014; Schwartz and Kipnis, 2005). The T_H_2 cytokine IL-4 plays a major role in cognition since IL-4^−/−^ mice display impaired spatial learning, which can be rescued by transplantation of IL-4–expressing T cells (Derecki et al., 2010). Furthermore, glia-derived TNFα was demonstrated to mediate homeostatic synaptic scaling (Stellwagen and Malenka, 2006), and in *Caenorhabditis elegans* neuronal IL-17 signaling was shown to play a regulatory role in escape behavior and associative learning (Flynn et al., 2020). In mice exposed to maternal immune deprivation, IL-17A was able to reverse deficits in social behavior (Reed et al., 2020).

Previously, we found that specifically T_H_2 lymphocytes, in contrast to pro-inflammatory T_H_1 cells, were able to improve corticospinal tract axon regeneration and functional recovery in traumatic injury by potentiating neurotrophin signaling (Walsh et al., 2015). Earlier reports on the beneficial role of exogenous IL-4 application within the CNS (Butti et al., 2008; Fenn et al., 2014) as well as the upregulation of brain-endogenous IL-4 after stroke (Zhao et al., 2015) led to the notion that this immune cytokine may serve neuroprotective or repair-related activities. In this context, studies focused mainly on beneficial effects through alternatively activated myeloid cells (Butovsky et al., 2006; Casella et al., 2018; Fenn et al., 2014; Francos-Quijorna et al., 2016). We recently discovered that IL-4Rα is involved in neuronal outgrowth and repair through a neuron-specific IL-4Rα signaling pathway in neuroinflammation (Hanuscheck et al., 2020; Vogelaar et al., 2018). Applied intrathecally or intranasally during the chronic phase of experimental autoimmune encephalomyelitis, IL-4 was able to ameliorate clinical symptoms through the repair of axon swellings and the induction of axon cytoskeleton remodeling.

Based on our previous findings of a fast direct IL-4R signaling pathway in neurons (Vogelaar et al., 2018), we hypothesized a homeostatic role in synaptic function. We now used neuron-specific IL-4Rα–deficient mice to elucidate the direct effects of the IL-4/IL-4R pathway on synaptic networks in neurons. Here, we demonstrate that synaptic vesicle recruitment is reduced when IL-4Rα is deficient. Long-term effects of IL-4Rα deficiency in adult mice include increased neuronal network activity and behavioral deficits. Thus, our data suggest that the IL-4 receptor pathway is relevant for the fine-tuning of synaptic transmission and maintaining homeostasis of the CNS.

## Results

### IL-4Rα–deficient neurons display altered gene expression profiles

**Figure 1.**
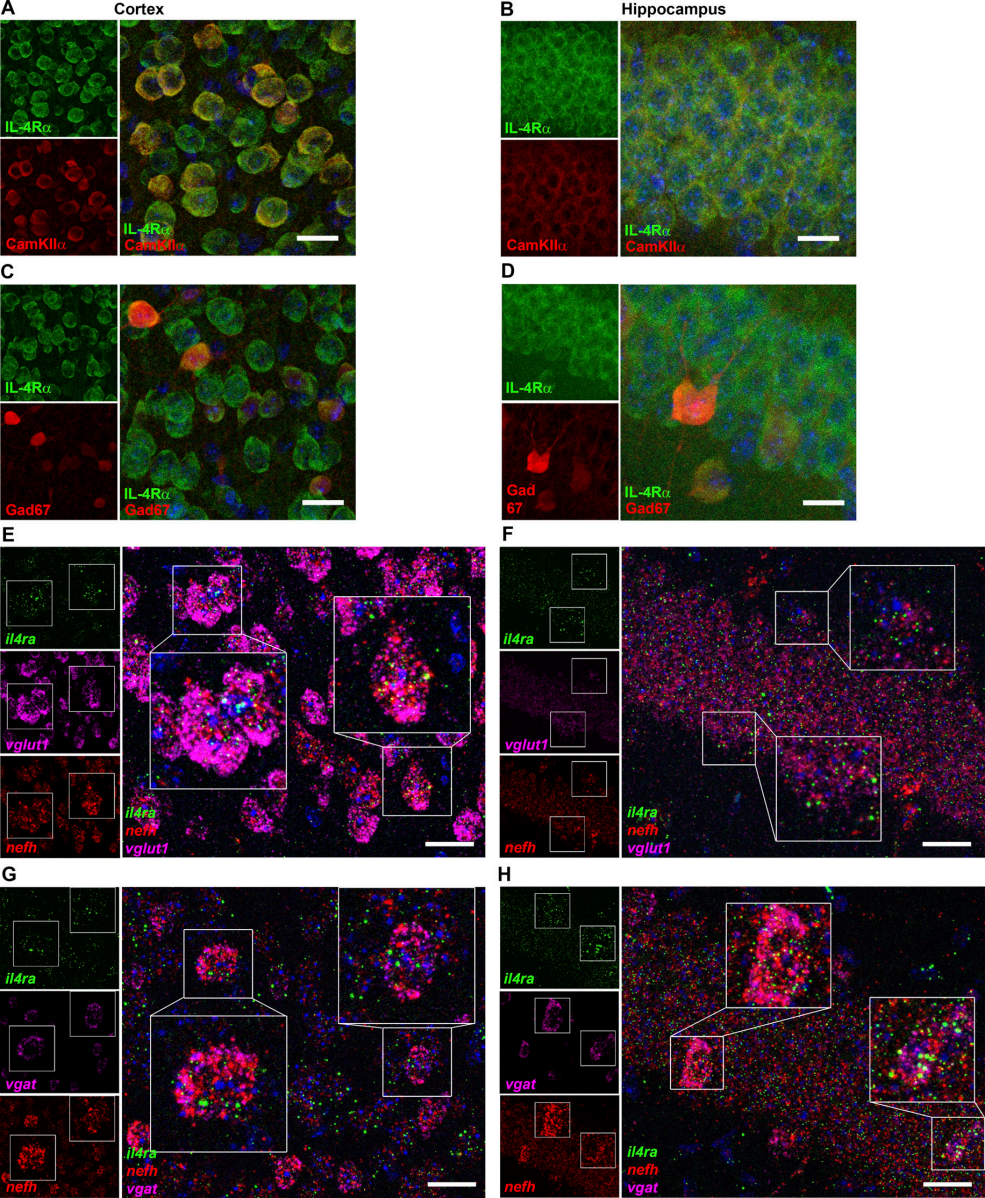
**IL-4Rα is expressed by both excitatory and inhibitory neurons. (A and B)** IHC for IL-4Rα (green) and CamkIIα (red) in the prefrontal cortex and hippocampus. **(C and D)** IHC for IL-4Rα (green) in glutamate decarboxylase 67-GFP neurons (Gad67, here in red) in prefrontal cortex and hippocampus (representative images of two independent experiments). **(E–H)** RNAscope for *il4ra* (green), *nefh* (red), *vglut1* (excitatory neurons, magenta), and *vgat* (inhibitory neurons, magenta) mRNA demonstrating coexpression of *il4ra* with excitatory and inhibitory neurons in cortex (left) and hippocampus (right) of adult C57Bl/6J mice (representative images of three independent experiments). Scale bars = 20 µm (A–H).

**Figure S1.**
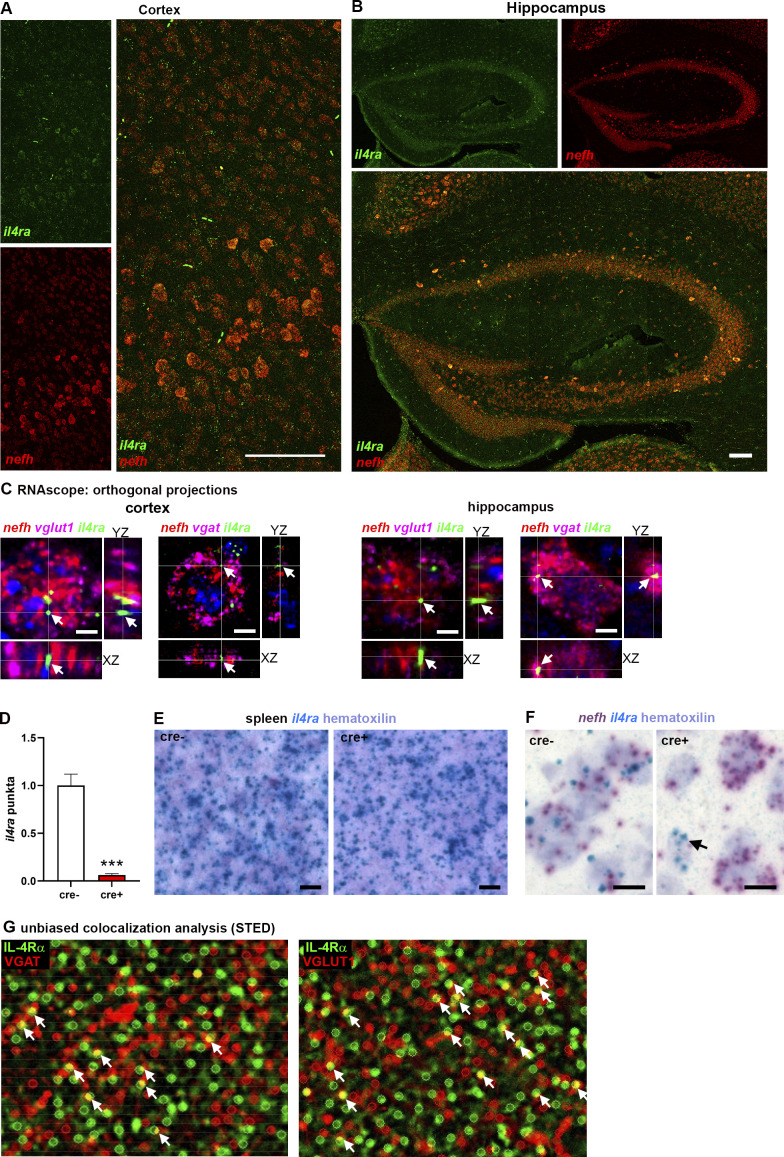
**Neuronal expression of IL-4Rα. (A and B)** RNAscope for *il4ra*, *nefh* on adult C57Bl/6J brains (representative images of three independent experiments) showing coexpression of *il4ra* (green) and *nefh* (red) in neurons in cortex, throughout the layers, and hippocampus, in CA regions and dentate gyrus. **(C)** Orthogonal projections of RNAscope signals in single neurons reveal coexpression of mRNA of *il4ra* (green), *nefh* (red), and *vglut1* (magenta, left panels) as well as *vgat* (magenta, right panels) in excitatory neurons of cortex and hippocampal CA1. **(D)** Quantification of *il4ra* exon 7 punctae showing abolishment of signal in il4ra^fl/fl^.Syn cre^+^ mice compared with cre^−^ littermates (*n* = 3, pooled from two independent experiments; mean ± SEM). **(E)** BaseScope on spleen shows non-neuronal cells in cre^+^ and cre^−^ littermates express *il4ra* mRNA, excluding off-target effects of cre (representative images of two independent experiments). **(F)** BaseScope analysis of *il4ra* and *nefh* mRNA reveals abolished *il4ra* mRNA signal in nefh^+^ neurons, but not in a non-neuronal cell (arrow) in cre^+^ littermates (representative images of two independent experiments). **(G)** Unbiased colocalization analysis of IL-4Rα (green) and VGAT (red, left panel), as well as VGLUT1 (red, right panel) for the quantification of colocalization (yellow, arrows; based on three biological replicates). Statistics: (D) Bayesian analysis, accuracy, *** > 95% (see Data S1).

**Figure 2.**
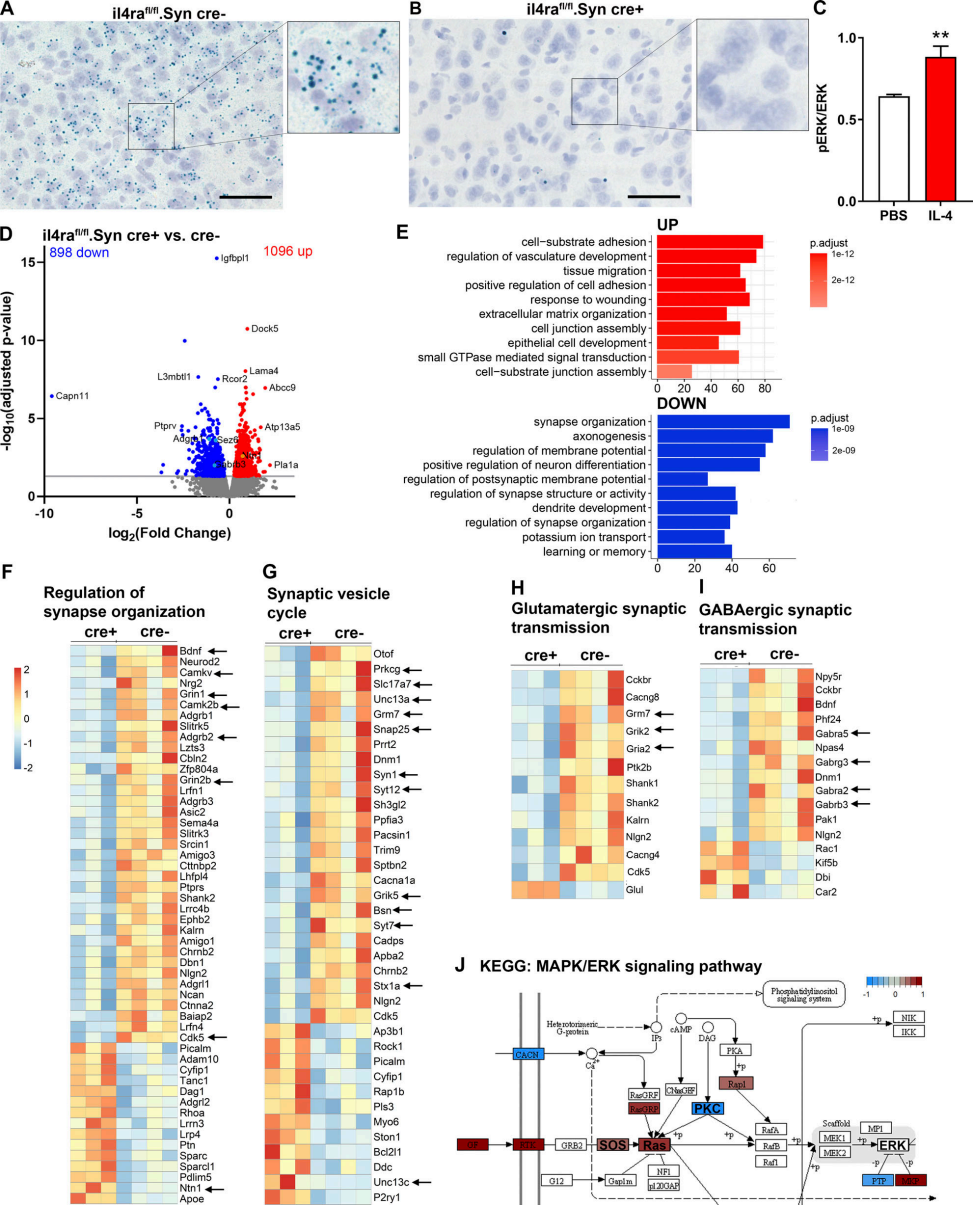
**IL-4Rα deficiency alters neuronal transcriptome. (A and B)** BaseScope for *il4ra *exon 7 mRNA shows widespread cortical expression of *il4ra*, which is (B) almost completely abolished in il4ra^fl/fl^.Syn cre^+^ littermates. Scale bars = 50 µm. Representative images of three independent experiments. **(C)** IL-4–induced phosphorylation of ERK in WT cortical neurons (quantification of representative of three independent experiments [*n* = 5 per group]; mean ± SEM). **(D)** RNAseq on il4ra^fl/fl^.Syn cre mice shows differential gene expression in cre^+^ neurons compared with cre^−^ controls (Volcano plot, *n* = 3–4 mice per genotype, 12- to 16-wk-old littermates). **(E)** Top 10 upregulated and downregulated GO terms. **(F and G)** Heatmaps of GO terms related to synapse organization (GO: 0050808) and synaptic vesicle cycle (GO: 0099504) showing the regulation of synaptic genes (arrows). **(H)** GO term for glutamatergic synaptic transmission (GO: 0051966) showing differential regulation of glutamatergic receptor components (arrows). **(I)** GO term for GABAergic synaptic transmission (GO: 0051932) showing differential expression of GABAergic receptors (arrows). **(J)** KEGG pathway for PKC and MAPK/ERK signaling. Statistics (C): Bayesian analysis, accuracy, ** >90% (see Data S1). Source data are available for this figure: SourceData F2.

Based on our previous observations of direct neuronal IL-4R signaling and expression of IL-4Rα on cortical and hippocampal neurons (Vogelaar et al., 2018), we now studied the expression in neuronal subtypes. Immunohistochemistry (IHC) for IL-4Rα protein together with the excitatory marker Ca^2+^-calmodulin–dependent protein kinase II alpha (CamkIIα; Fig. 1, A and B) and glutamate decarboxylase 67 (Gad67; Fig. 1, C and D), which is expressed in inhibitory neurons, showed IL-4Rα protein expression in both excitatory and inhibitory neurons in the cortex and hippocampus. To confirm IL-4Rα expression at the RNA level, we performed RNAscope for *il4ra* mRNA and found coexpression with *neurofilament heavy chain* (*nefh*; Figs. 1, E–H; and S1, A and B), *vesicular glutamate transporter 1* (*vglut*; Fig. 1, E and F), and *vesicular gaba transporter* (*vgat*; Fig. 1, G and H). Coexpression of *il4ra* mRNA with these molecules was verified via orthogonal projections (Fig. S1 C). Using probes exclusively for exon 7, one of the excised exons causing the functional deficiency of the used transgenic animals (Herbert et al., 2004), neuronal IL-4Rα was proven to be abolished in il4ra^fl/fl^.Syn cre^+^ brain (Figs. 2, A and B; and S1 D). Positive staining in both cre^+^ and cre^−^ spleen tissue confirmed neuronal specificity of the deletion and ruled out off-target cre-recombination (Fig. S1 E). In addition, using a combination of BaseScope probes for *nefh* and *il4ra* exon 7, we were able to demonstrate *il4ra* deficiency of *nefh*^*+*^ neurons, with persistent expression in non-neuronal cells (Fig. S1 F).

After having previously identified a neuronal IL-4Rα signaling pathway via IRS1, PI3K, PKCγ, and GAP-43 (Hanuscheck et al., 2020; Vogelaar et al., 2018), we now found that IL-4 was able to induce ERK phosphorylation in neurons (Fig. 2 C). Since ERK is known to activate transcriptional programs involved in synaptic transmission (Bluthgen et al., 2017; Thomas and Huganir, 2004), we performed RNA sequencing (RNAseq) analysis of il4ra^fl/fl^.Syn cre^+^/cre^−^ neurons and indeed found 1,994 differentially expressed genes, of which 898 were significantly downregulated and 1,096 were significantly upregulated (Fig. 2 D). Gene ontology (GO) analysis revealed that cre^+^ mice displayed upregulated genes associated with GO terms for cell migration and adhesion. We observed a substantial downregulation of synapse-related gene expression including genes involved in synaptic processes. Other downregulated GO terms included “axonogenesis,” providing additional evidence for the presynaptic influence of IL-4 (Fig. 2 E). Of particular interest is the GO term “regulation of synapse organization” (Fig. 2 F), in which genes such as *adhesion G protein-coupled receptor B1* (*Adgrb1*), *bdnf*, *cyclin-dependent-like kinase 5* (*cdk5*), and signaling molecules such as *calcium calmodulin-dependent protein kinase II beta* (*camk2b*) and *CaM kinase-like vesicle-associated* (*camkv*), as well as the NMDA receptor subunits *glutamate ionotropic receptor NMDA type subunit 1* and *2b* (*grin1* and *-2b*) were all downregulated in cre^+^ neurons, indicating modification of synaptic plasticity, signaling, and neurotransmission. In contrast, the guidance molecule *Netrin 1* (*ntn1*) was upregulated. Importantly, as becomes clear from the GO term “synaptic vesicle cycle” (Fig. 2 G), genes directly involved in synapse assembly, signaling, and vesicle release, like *bassoon* (*bsn*), *munc13-1* (*unc13a*), *protein kinase C gamma* (*prkcg*), *synapsin1* (*syn1*), *synaptosomal-associated protein*, *25 kD* (*SNAP-25*), *synaptotagmin* (*syt*) *7* and *12*, *syntaxin1a* (*stx1a*), and *solute carrier family 17 member 7* (*slc17a7*, also known as VGLUT1), were downregulated in cre^+^ neurons. In addition to the glutamatergic receptor genes in this GO term, such as *glutamate receptor ionotropic kainate 5* (*grik5*) and *metabotropic glutamate receptor 7* (*grm7)*, analysis of genes annotated with the GO terms “glutamatergic synaptic transmission” (Fig. 2 H) and “GABAergic synaptic transmission” (Fig. 2 I) revealed downregulation of further genes involved in both glutamatergic as well as GABergic synaptic transmission including *glutamate ionotropic receptor AMPA type subunit 2* (*gria2*) and *glutamatergic ionotropic receptor kainate type subunit 2* (*grik2*), as well as four GABA receptor subunits, namely *gamma-aminobutyric acid type a receptor subunit alpha2* and *-5*, *beta3*, and *gamma3* (*gabra2*, *-a5*, *-b3*, and *-g3*). These data confirm the functional involvement of IL-4Rα signaling in both excitatory and inhibitory neurons. To understand the consequences of neuronal IL-4Rα deficiency on molecular pathways, we performed Kyoto Encyclopedia of Genes and Genomes (KEGG) analysis, thereby revealing, among others, altered regulation of key signaling molecules including PKC and components of the MAPK/ERK pathway (Fig. 2 J).

**Figure S2.**
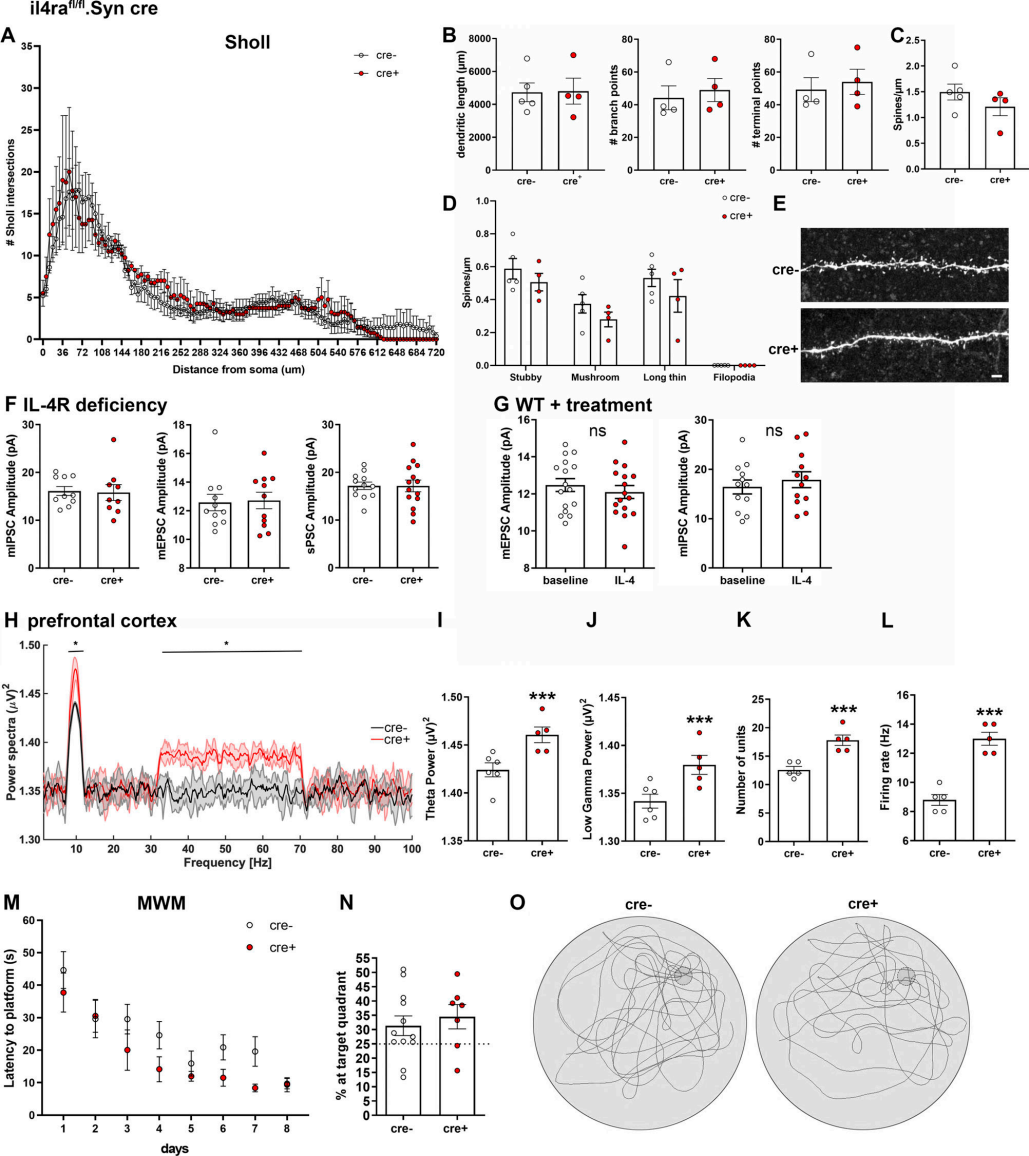
**Neuronal IL-4R deficiency has no effects on dendrite morphology, PSC amplitudes, and Morris water maze.** Analysis of biocytin-filled patched CA1 neurons of P30 il4ra^fl/fl^.Syn cre mice (cre^−^ *n* = 5, cre^+^ *n* = 4, littermates, pooled data from two independent experiments). **(A and B)** Sholl analysis reveals no differences in number of Sholl intersections, nor in (B) dendritic length, branch points, or terminal points. **(C and D)** Spine analysis shows no significant difference in spine density, nor in (D) spine morphology. **(E)** Exemplary images of spines of secondary dendrite of cre^+^ and cre^−^ mice. Scale bar = 2 µm. **(F)** Amplitudes of mEPSCs, mIPSCs, and sPSCs were unchanged in il4ra^fl/fl^.Syn cre mice (9–10 neurons from at least three littermates per genotype, representative data from two independent experiments). **(G)** Amplitudes of mEPSCs and mIPSCs were unchanged after treatment with IL-4 (10–14 neurons from at least three mice per treatment, representative data from two independent experiments). **(H–L)** In vivo recordings of LFPs in mPFC (two independent experiments) show an increase in θ and low γ waves in il4ra.^fl/fl^.Syn cre^+^ mice compared with cre^−^ littermates (*n* = 5 each, male, 12- to 16-wk-old), accompanied by increased (K) number of units firing and (L) firing rate. **(M)** Morris water maze (MWM) test (two independent experiments) showed no difference in learning between cre^+^ and cre^−^ littermates. **(N)** Mice spend equal times in the target zone at final test in MWM. **(O)** Representative graphs of swimming patterns at target test. Plots (B–D, F, G, and I–N) depict mean ± SEM. Statistics: (A, D, H, and M) two-way ANOVA, * P < 0.05; (B–C, F–G, I–L, and N) Bayesian analysis, accuracy, * >80%, ** >90%, *** >95% (see Data S1).

Taken together, these data indicate that the absence of functional IL-4Rα in mouse neurons leads to massive changes in transcript levels of proteins involved in the function of synaptic connections. It should already be noted that these changes cannot be explained by developmental abnormalities of the Syn cre transgenics (see Fig. S2, A–E).

### Gene expression profiles in human neurons

**Figure 3.**
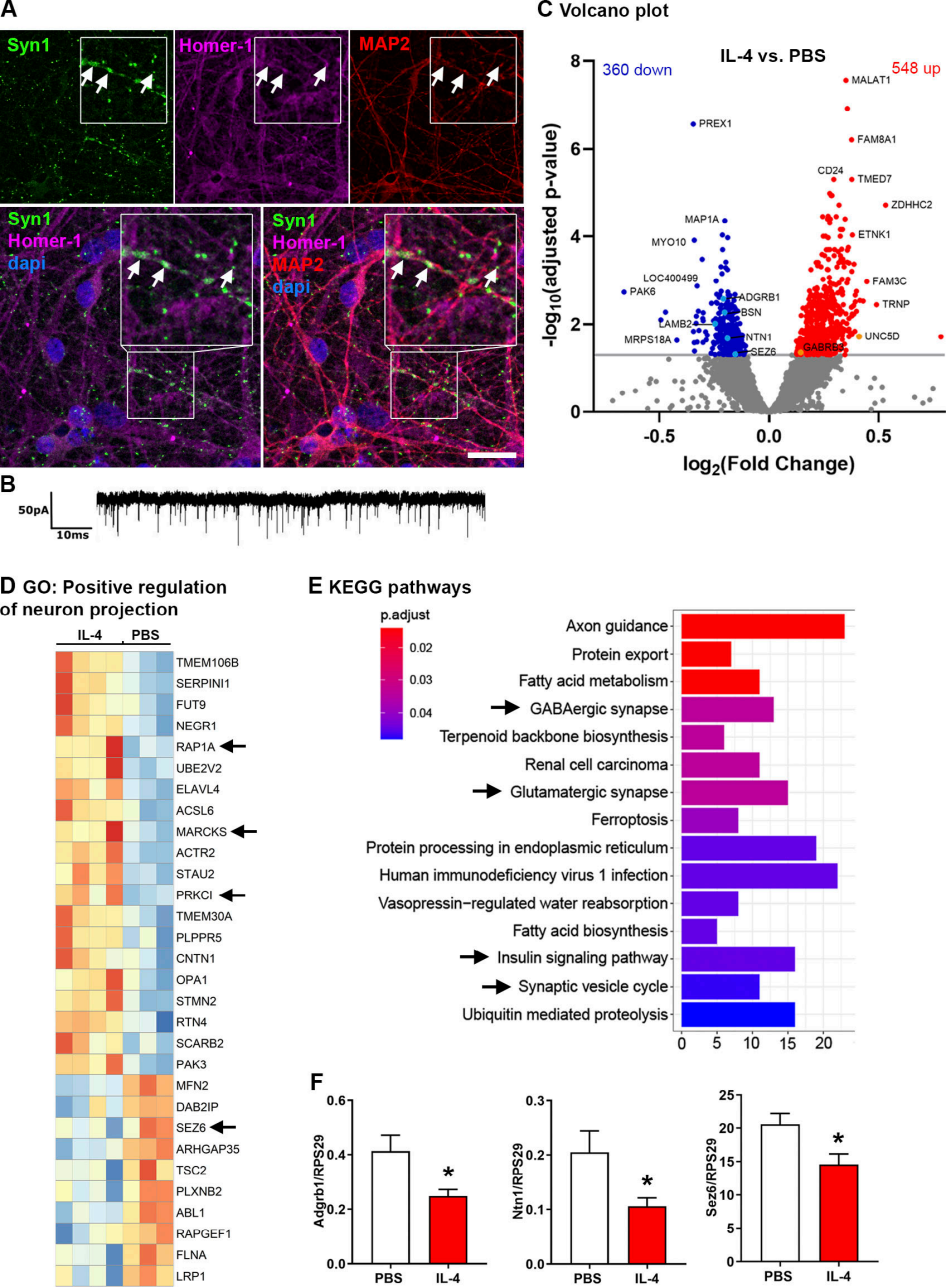
**Human neurons build synapses and alter transcriptional programs in response to IL-4. (A)** Immunocytochemistry for Syn1 (green), Homer-1 (magenta), MAP2 (red), and Dapi (blue), showing human neurons build synapses (arrows) at 35 d in vitro. Scale bar = 25 µm. **(B)** Patched human neurons displaying spontaneous electrical activity. **(C)** RNAseq on human neurons treated for 7 d with IL-4 (*n* = 4) or PBS (*n* = 3): Volcano plot of differentially regulated genes. **(D)** GO term for positive regulation of neuron projection (GO: 0010976) showing, among others, signaling molecules (arrows). **(E)** KEGG pathway analysis showing regulation of relevant synaptic pathways (arrows). **(F)** RT-qPCR on murine IL-4–treated neurons. Representative quantifications of two independent experiments (PBS, *n* = 3; IL-4, *n* = 6); mean ± SEM. Statistics (F): Bayesian analysis, accuracy, * >80% (see Data S1).

To investigate the human neuronal system, we differentiated human neurons from induced pluripotent stem cell (iPSC)–derived neural progenitor cells (NPCs) and compared IL-4 treatment with PBS-treated control neurons using RNAseq. These neurons created elaborate networks with synapses (Fig. 3 A) and were electrophysiologically active (Fig. 3 B). We observed a total of 908 differentially regulated genes; 360 showed decreased expression while 548 genes were significantly increased (Fig. 3 C). Upregulated genes included *γ-aminobutyric acid receptor subunit β-3* (*Gabrb3*) and *unc5d* (*netrin receptor*), whereas *Adgrb1*, *Bsn*, *laminin subunit β 2* (*Lamb2*), *netrin 1* (*Ntn1*), and *seizure-related 6 homolog* (*Sez6*) were downregulated (Fig. 3 C). Among the significantly upregulated GO terms was “positive regulation of neuron projection,” which includes the signaling molecules *protein kinase c iota* (*prkci*) and *ras-related protein 1a* (*rap1a*), as well as the known synaptic PKC target *myristoylated alanine-rich c-kinase substrate* (*MARCKS*; Calabrese and Halpain, 2005; Fig. 3 D). KEGG pathway analysis revealed that molecules in the insulin-signaling pathway and synaptic-vesicle cycle as well as pathways in glutamatergic and GABAergic synapses were regulated (Fig. 3 E).

Notably, murine dissociated neurons, treated with IL-4 in the same way as the human neurons, also revealed the downregulation of genes such as *Adgrb1*, *Ntn1*, and *Sez6* (Fig. 3 F and Data S1), thus confirming overlapping effects of IL-4 between species. These molecules are known for their role in synaptic functions: Ntn1 is involved in synapse assembly (Goldman et al., 2013), Adgrb1 induces clustering of VGLUT1 (Tu et al., 2018), and Sez6 affects neuronal excitability (Gunnersen et al., 2007).

In conclusion, IL-4 treatment of human iPSC-derived neurons leads to the regulation of synaptic components, signaling molecules, and genes involved in synaptogenesis, which is consistent with the murine data, indicating a role of the IL-4Rα pathway in synapse formation and signaling.

### IL-4Rα localizes to presynaptic terminals and impacts synaptic vesicle release

**Figure 4.**
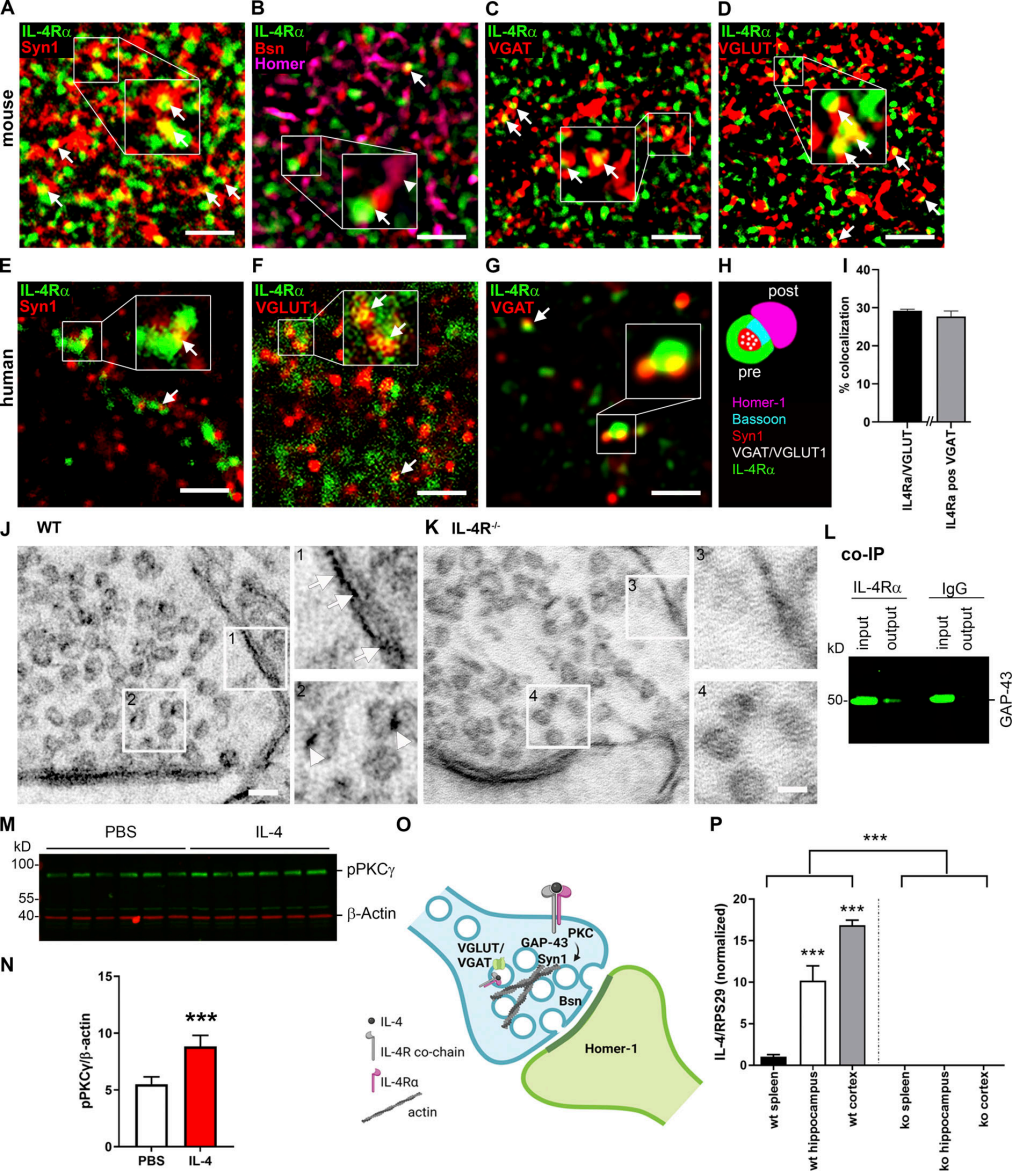
**IL-4Rα pathway in the presynapse. (A–D)** Exemplary images of STED microscopy for IL-4Rα and synaptic markers in hippocampal stratum radiatum of adult mice. Representative images of two independent experiments. Boxed areas are enlarged for improved visualization. **(A)** IL-4Rα (green) colocalizes (arrows) with presynaptic Syn1 (red). **(B)** IL-4Rα is in close proximity (arrow) to the active zone protein Bsn (red), but not to Homer-1 (magenta, arrowhead). **(C)** Inhibitory synapses marked by VGAT (red) also express IL-4Rα (arrows). **(D)** IL-4Rα expression in excitatory synapses marked by VGLUT1 (red, arrows). **(E–G)** STED microscopy in human postmortem brain tissue shows overlap (arrows) of IL-4Rα (green) with (E) Syn1 (red), (F) VGLUT1 (red), and (G) VGAT (red). **(H)** Schematic summary of presynaptic IL-4Rα localization. **(I)** Quantification of colocalization of IL-4Rα with VGLUT and VGAT in murine stratum radiatum. **(J)** ImmunoEM of synapse in stratum radiatum of Balb/C WT (The Jackson Laboratory) showing expression on the membrane (arrows) and vesicles (arrowhead). **(K)** Expression is abolished in Balb/C.il4ra^−/−^ synapse. Boxed areas (1–4) are enlarged for improved visualization. Representative images of three independent experiments. **(L)** Co-IP with anti–IL-4Rα or isotype IgG control on synaptosomes shows presence of GAP-43 (∼50 kD) in the presynaptic signaling pathway. Input represents total synaptosome lysate; output corresponds to the co-IP product; GAP-43 was absent in isotype control. Based on three independent experiments. **(M)** Representative Western blot (three independent experiments) of pPKCγ (∼90 kD) and β-actin (∼40 kD) for PBS and IL-4–treated ultrapure synaptosomes. **(N)** Quantification shows induction of PKCγ phosphorylation by IL-4 (*n* = 6) compared to PBS (*n* = 5). **(O)** Schematic representation of IL-4R signaling pathway in presynapse. **(P)** Quantification of IL-4 expression relative to RPS29, normalized to spleen reveals IL-4 expression in hippocampus and cortex, abolished in IL-4 KO (*n* = 4 per group). Scale bars = 1 µm (A–G); 50 nm (J and K). Plots (I, N, and P) depict mean ± SEM. Statistics: Bayesian analysis, accuracy, * >80%, ** >90%, *** >95% (see Data S1). Source data are available for this figure: SourceData F4.

Due to the discovered gene expression profile indicating involvement of IL-4/IL-4Rα signaling in the regulation of synaptic genes, we next investigated the synaptic localization of IL-4Rα. Using stimulated emission depletion (STED) microscopy, we analyzed colocalization of IL-4Rα protein with markers for different synaptic compartments in the stratum radiatum of the hippocampal CA1 region. We observed abundant colocalization of IL-4Rα with Syn1 in the presynaptic compartment (Fig. 4 A), as well as IL-4Rα in close proximity to Bassoon, a core component of the presynaptic active zone, but not to the postsynaptic protein Homer-1 (Fig. 4 B). IL-4Rα was observed in both inhibitory and excitatory synapses as shown by colocalization with the markers VGAT and VGLUT1, respectively (Fig. 4, C and D). In line with this, STED microscopy on human postmortem material demonstrated IL-4Rα expression overlapping with Syn1, suggesting similar presynaptic localization of IL-4Rα in the human cortex (Fig. 4 E). In human synapses, we again detected IL-4Rα colocalization with VGLUT1 and VGAT (Fig. 4, F and G). Thus, as summarized in Fig. 4 H, IL-4Rα signals were found in the presynaptic compartment of both glutamatergic and GABAergic synapses. Quantification of IL-4Rα signals by unbiased machine learning revealed that among hippocampal synapses ∼29.2% of IL-4Rα was colocalized with VGLUT1, whereas ∼27.6% was colocalized with VGAT (Figs. 4 I and S1 G). For further fine structural analysis, we performed immuno-electron microscopy (immunoEM) in wild-type mice showing localization of IL-4Rα on the presynaptic membrane (Fig. 4 J, arrows), and interestingly, also on synaptic vesicles within the presynaptic terminal (Fig. 4 J, arrowheads). The absence of signal in il4ra^−/−^ synapses confirmed the specificity of the antibody (Fig. 4 K). Co-immunoprecipitation (co-IP) on synaptosomes using anti–IL-4Rα as bait revealed GAP-43, which is known to be involved in the regulation of the presynaptic actin cytoskeleton (Angliker and Ruegg, 2013), as the interaction partner in the synapse (Fig. 4 L). Taken together, these data suggest a presynaptic localization of IL-4Rα and its signaling pathway.

Furthermore, when compared to PBS control, treatment of synaptosomes with IL-4 revealed induction of phosphorylation of PKCγ (Fig. 4, M and N; and Data S1), a molecule from the IL-4R pathway we previously identified as relevant for axonal outgrowth (Vogelaar et al., 2018). These data demonstrate that the IL-4R pathway is active in the presynaptic compartment (Fig. 4 O). In light of these findings, we hypothesize that IL-4Rα signaling may play a role in vesicle recruitment in synaptic function. The prerequisite for such a function is the presence of IL-4 in the brain. Accordingly, we found that *il4* mRNA is present in hippocampus and cortex in homeostasis, at even higher levels than in spleen and absent in IL-4 KO (Fig. 4 P and Data S1).

**Figure 5.**
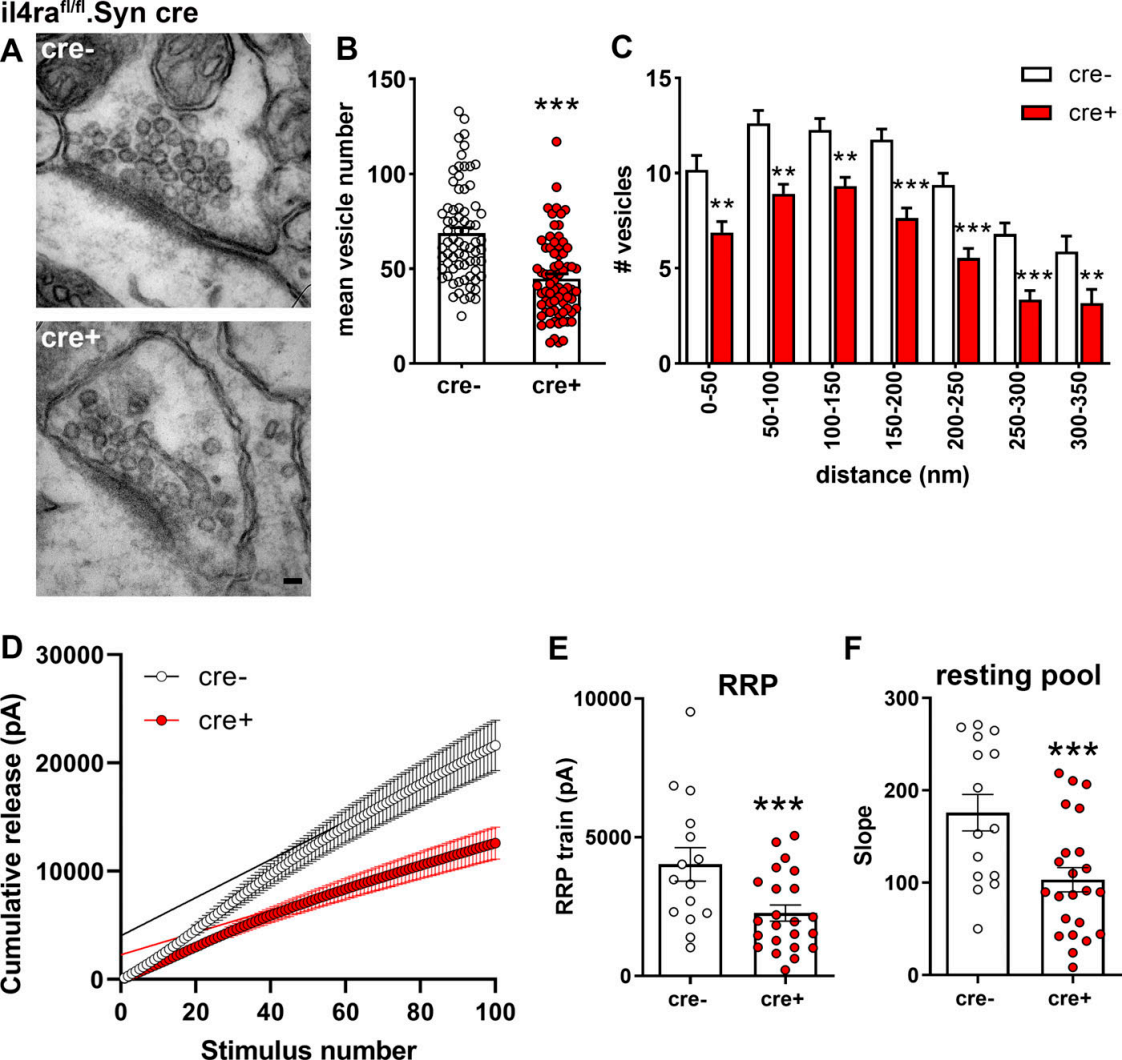
**Functional consequences of IL-4Rα deficiency on synaptic vesicle pools. (A)** Representative EM images from two independent experiments of synapses in hippocampal stratum radiatum of il4rafl/fl.Syn cre^+^ and cre^−^ littermates. **(B)** Quantification of vesicle numbers (cre^−^ *n* = 6, 72 synapses; cre^+^ *n* = 6, 73 synapses) shows reduction in cre^+^ mice compared to cre^−^ controls. **(C)** Quantification of vesicle numbers at defined distances from the active zone. **(D–F)** Cumulative EPSC amplitudes in patched CA1 neurons (15–22 neurons from at least three littermates per genotype) after train stimulations of 100 pulses at 20 Hz show a reduction of the (E) RRP (y-intercept) and (F) resting pool (slope) of synaptic vesicles. Pooled data from two independent experiments. Scale bar = 50 nm (A). Plots (E and F) depict mean ± SEM. Statistics: (C) two-way ANOVA, ** P < 0.01, *** P < 0.001; (B, E, and F) Bayesian analysis, accuracy, * >80%, ** >90%, *** >95% (see Data S1).

Based on our findings on presynaptic IL-4Rα signaling, we next analyzed the effect of IL-4Rα deficiency at the fine structural level by electron microscopy (EM) on the synapses of il4ra^fl/fl^.Syn cre mice (Fig. 5 A). Here, we used the hippocampus for the evaluation of basic properties of cortical neurons and found a reduced number of total vesicles in IL-4Rα–deficient synapses as compared to cre^−^ controls (Fig. 5 B and Data S1). Analysis of vesicle numbers at defined distance bins from the active zone revealed a reduction in vesicles at the active zone as well as a decrease in the reserve vesicle pool, which is located further away from the active zone (Fig. 5 C and Data S1). To study the functional consequences of the decreased vesicle numbers and altered distribution observed by EM, we performed electrophysiology. We measured vesicle release by train stimulation (100 pulses at 20 Hz) of the Schaffer collateral/commissural fibers in the stratum radiatum and simultaneous recording of excitatory postsynaptic currents (EPSCs) from CA1 neurons. This analysis allows the estimation of the readily releasable pool (RRP) and resting pool size (Neher, 2015; Schneggenburger et al., 1999; Thanawala and Regehr, 2013). A cumulative plot of the peak amplitude versus stimulus number was drawn (Fig. 5 D). The y-intercept of the back extrapolation of a linear fit of the 90–100th pulses was smaller in cre^+^ animals than in cre^−^ littermates (2,269.7 ± 284.9 vs. 4,025.6 ± 585.5 pA, mean ± SEM, 97.4% accuracy), indicating that IL-4Rα deficiency reduced the effective RRP size (Fig. 5 E and Data S1). In addition, the replenishment rate of new neurotransmitter vesicles from the resting vesicle pool was reduced in cre^+^ mice, as evidenced by the reduced slope of the linear fit (103.3 ± 12.7 vs. 175.9 ± 19.1, mean ± SEM, 99.6% accuracy; Fig. 5 F and Data S1). Importantly, patched cells were filled with biocytin and stained with Alexa-488–conjugated streptavidin for Sholl and spine analysis, which revealed no differences in dendritic length, branching, or terminals. Spine density and subtype were also unchanged (Fig. S2, A–E). The absence of structural differences and the preserved synaptic density together with the unaltered spine maturation stages strongly indicates that the observed electrophysiological phenotypes are not due to alterations in embryonic development.

Collectively, these data show that neuronal IL-4Rα deficiency influences synaptic transmission at the level of the presynapse leading to a reduction in recruitment of vesicles.

### IL-4/IL-4Rα signaling plays a role in synaptic activity

**Figure 6.**
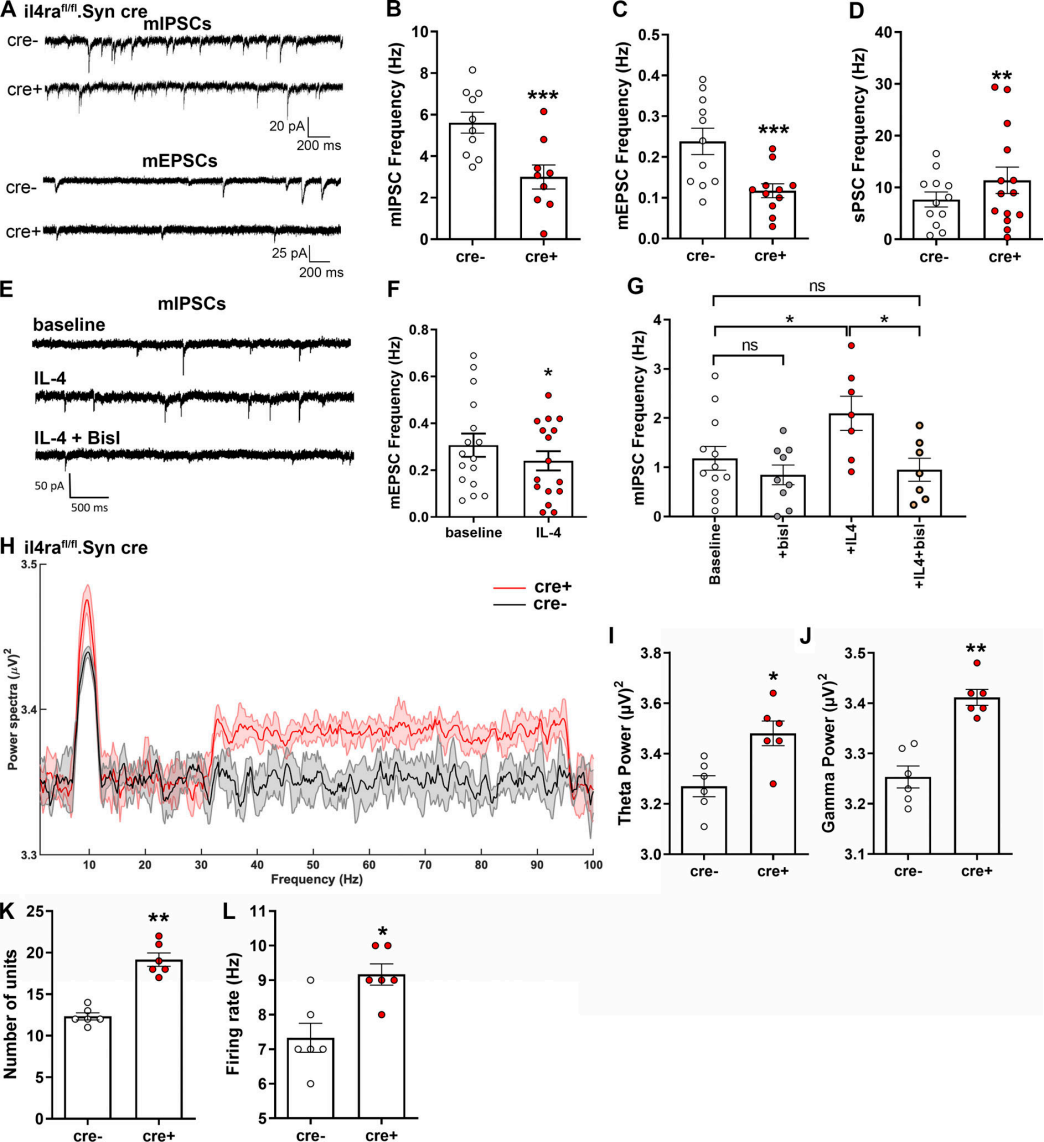
**Neuronal IL-4R deficiency results in altered electrophysiological properties due to differences in synaptic vesicle pools.** Patch clamp recordings in CA1 hippocampal neurons in P29-35 il4ra^fl/fl^.Syn cre mice (9–10 neurons from at least three littermates per genotype [A–G]; representative data from two independent experiments). Plots depict mean ± SEM, individual data points (neurons) are provided. **(A)** Exemplary traces of mIPSCs and mEPSCs. **(B and C)** mIPSCs (B) and mEPSCs (C) both show reduced frequency in cre^+^ mice compared to cre^−^ controls. **(D)** sPSCs show increased spontaneous activity of cre^+^ neurons. **(E)** Exemplary traces of mIPSC recordings in CA1 neurons at baseline, after IL-4 treatment, and with co-incubation of IL-4 and BisI (10–14 neurons from at least three mice per treatment). **(F)** Quantification of frequencies of mEPSCs. **(G)** Quantification of mIPSCs in CA1 neurons at baseline, after treatment with BisI or IL-4 and IL-4 + BisI. **(H–L)** In vivo recordings of LFPs in hippocampus show an increase in θ and γ waves in il4ra^fl/fl^.Syn cre^+^ mice compared with cre^−^ littermates (*n* = 6 each, male, 12- to 16-wk-old), accompanied by increased (K) number of units firing and (L) firing rate. Statistics: (B–D and I–L) Bayesian analysis, accuracy, * >80%, ** >90%, *** >95% (see Data S1); (F) paired *t* test, * P < 0.05; (G) one-way ANOVA with Tukey’s multiple comparisons test, * P < 0.05.

To evaluate the consequences of the synaptic phenotype upon IL-4Rα deficiency, we next analyzed miniature postsynaptic potentials (mEPSCs and miniature inhibitory postsynaptic potentials [mIPSCs]; Fig. 6, A–D; and Data S1). Here, we performed patch clamping of hippocampal neurons in slices of il4ra^fl/fl^.Syn cre mice and found decreased frequencies of both types of postsynaptic currents in IL-4Rα–deficient neurons (mIPSCs 3.002 ± 0.5777 vs. 5.615 ± 0.5036 Hz, mean ± SEM, 99.5% accuracy and mEPSCs 0.1173 ± 0.0169 vs. 0.2382 ± 0.0323 Hz, mean + SEM, 99.8% accuracy; Fig. 6, B and C; and Data S1). These changes in mEPSC and mIPSC frequencies are consistent with the reduced RRP. The amplitudes of all postsynaptic currents were unchanged, indicating that the postsynaptic mechanisms did not play a role (Fig. S2, F and G).

**Figure S3.**
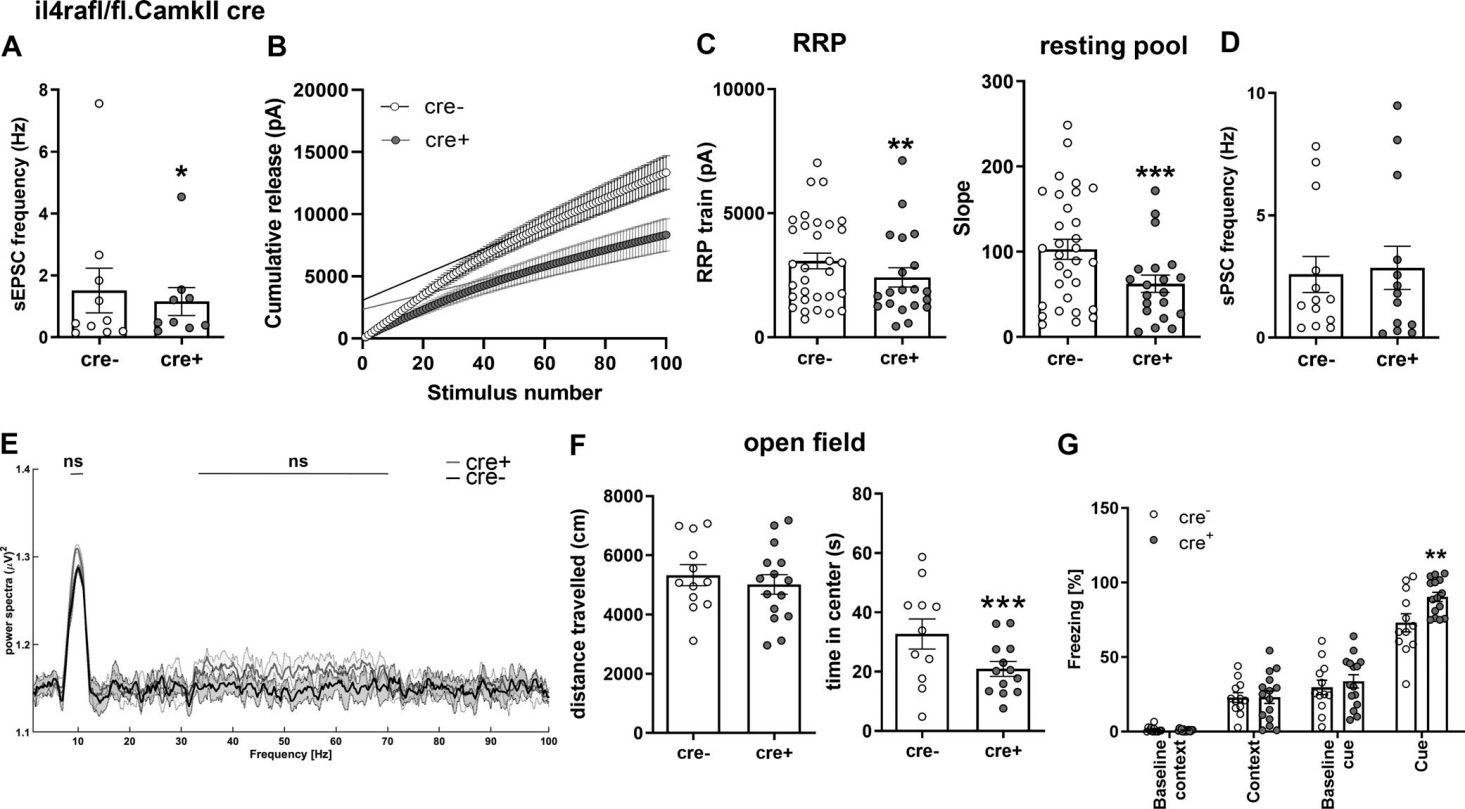
**ll4ra**^**fl/fl**^**.CamKIIα cre mice show mild phenotypes.** Electrophysiology and behavior of excitatory neuronal IL-4Rα–deficient mice. **(A)** sEPSCs are decreased in cre^+^ mice (9–10 neurons from at least three littermates per genotype). **(B and C)** Train stimulation (21–32 neurons from at least three littermates per genotype) shows (C) a reduction of both the RRP and the resting vesicle pool. **(D)** sPSCs are unaltered (9–10 neurons from at least three littermates per genotype). **(E)** In vivo LFP recordings show no significant difference in θ and low γ power (*n* = 5 littermates per genotype). **(F)** Behavioral analysis (cre^−^ *n* = 12, cre^+^ *n* = 15, littermates, representative results of two independent experiments) showed that (F) Cre^+^ mice displayed no difference in open field locomotion, but spent less time in the open field center. **(G)** Cre^+^ mice displayed higher levels of freezing in response to the conditioned cue. Plots (A–D, F, and G) depict mean ± SEM. Statistics: (A and C–F) Bayesian analysis, accuracy, * >80%, ** >90%, *** >95% (see Data S1); (G) two-way ANOVA, ** P < 0.01.

However, when examining the spontaneous postsynaptic currents (sPSCs), which reflect the net result of excitatory and inhibitory currents at the neuron level, we found that these were increased in cre^+^ mice as compared with cre^−^ littermates (11.40 ± 2.558 vs. 7.688 ± 1.447 mean ± SEM, 87.0% accuracy; Fig. 6 D and Data S1). This indicates that the net effect of altered presynaptic function eventually resulted in a higher excitatory drive, which is due to the predominant influence of the inhibitory plasticity on neuronal excitability as reported previously (Carvalho and Buonomano, 2009). Negative effects on excitatory transmission (reduction of spontaneous EPSCs [sEPSCs]) in il4ra^fl/fl^.CamKIIα cre^+^ mice (Fig. S3 A), where IL-4Rα deficiency is restricted to excitatory cortical neurons, support this interpretation. In this strain, we also found a change in the vesicle pools after train stimulation, pointing to an altered synaptic release in excitatory synapses under continuous stimulation (Fig. S3, B and C). This modification of synaptic transmission in excitatory synapses of il4ra^fl/fl^.CamKIIα cre^+^ mice did not lead to changes at the network level, as shown by unaltered sPSCs (Fig. S3 D).

Based on the above-described effects of IL-4Rα deficiency on neuronal activity, we questioned whether acute IL-4 treatment is capable of fine-tuning synaptic transmission in wild-type mice, i.e., during homeostasis (Fig. 6, E–G; and Data S1). Interestingly, baseline recorded neurons incubated with IL-4 for 10 min displayed a decreased mEPSC frequency (0.2400 ± 0.04129 vs. 0.3069 ± 0.04955 Hz, mean ± SEM, 83.7% accuracy; Fig. 6 F) and an increased mIPSC frequency (2.098 ± 0.3462 vs. 1.183 ± 0.2404 Hz, mean ± SEM, 95.7% accuracy; Fig. 6 G and Data S1) when recording was continued in the same cells. Again, amplitudes were unchanged, so IL-4 had no effect on the postsynapse (Fig. S2 F). Thus, acute IL-4 treatment led to an overall increase in inhibitory activity. In line with the induction of PKC phosphorylation in synaptosomes (Fig. 4 N), the IL-4–induced increase in the mIPSC frequencies could be abolished upon PKC inhibition by the application of bisindlolylmaleimide I (BisI; mIPSCs: 2.098 ± 0.3462 vs. 0.9524 ± 0.2324 Hz, mean ± SEM, 97.4% accuracy; Fig. 6 G and Data S1). This proves that presynaptic IL-4R signaling through PKC directly affects inhibitory synaptic responses.

### IL-4Rα deficiency leads to increased synchronized network activity

To investigate the in vivo consequences of IL-4Rα deficiency on the network level, we performed field recordings in the hippocampus of cre^+^ and cre^−^ littermates. Here, the power spectra of θ and γ oscillations were increased in pan-neuronal IL-4Rα–deficient mice (Fig. 6, H–J; and Data S1). Moreover, cre^+^ mice displayed a higher number of active neurons (Fig. 6 K and Data S1), which fired at a higher frequency when compared to cre^−^ controls (Fig. 6 L and Data S1). The same holds true for prefrontal cortical local field potentials (Fig. S2, H–L). In the il4ra^fl/fl^.CamKIIα cre line, in which IL-4Rα deficiency is restricted to excitatory cortical neurons, field recordings showed no changes in the power spectra of θ and low-frequency γ oscillations (Fig. S3 E).

In sum, in vivo and ex vivo results strongly suggest that pan-neuronal IL-4Rα deficiency within the CNS leads to higher neuronal excitability and shifts neuronal networks toward higher synchronized activity.

### Behavioral analysis shows an exploratory and anxiety-related learning behavioral phenotype

**Figure 7.**
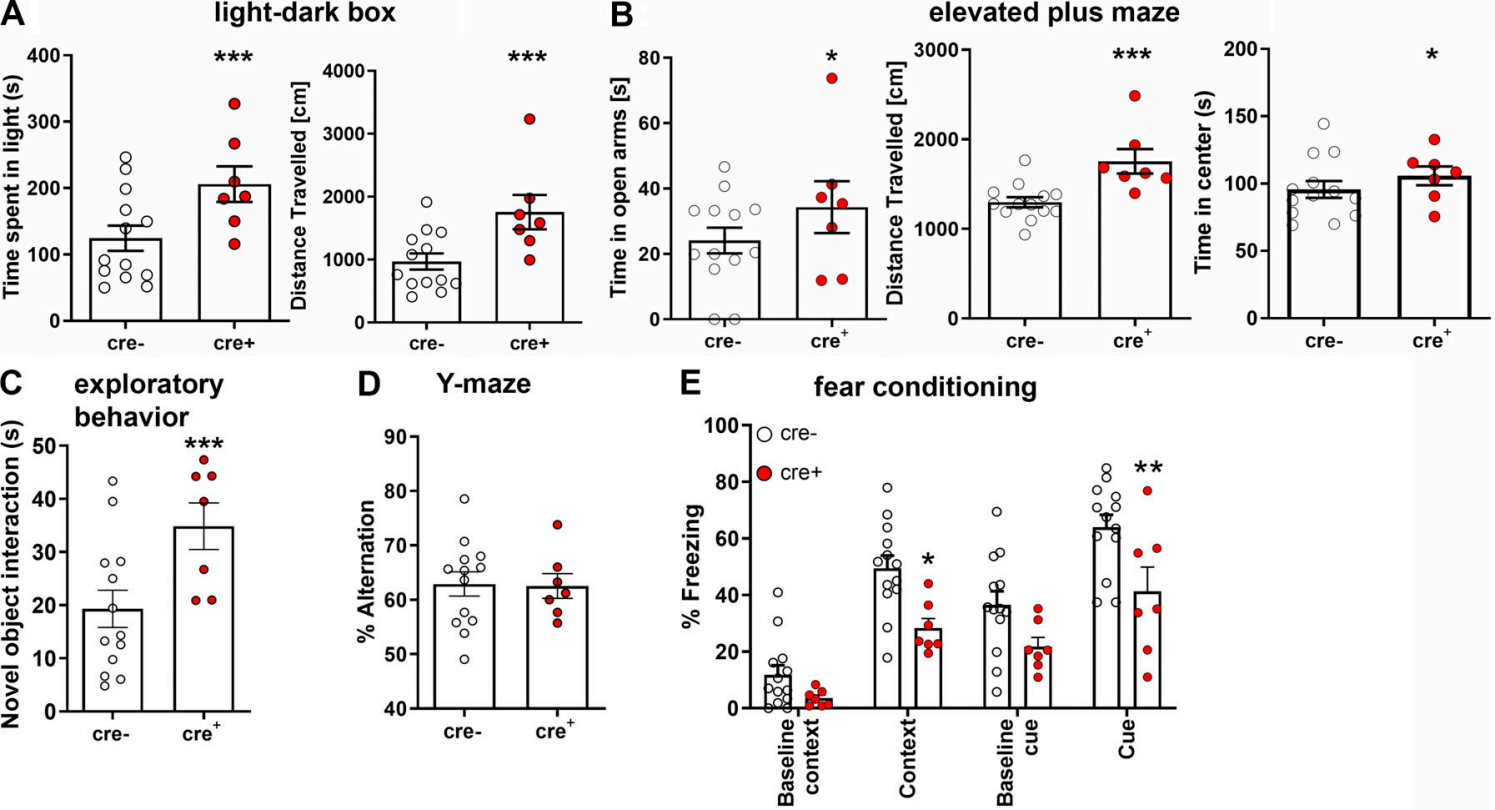
**Neuronal IL-4R deficiency results in altered neuronal networks and behavioral deficits. (A)** Behavioral analysis of 9- to 19-wk-old male il4ra^fl/fl^.Syn cre mice (cre^+^ *n* = 7, cre^−^ *n* = 13, littermates, representative data from two independent experiments) shows increased distance traveled in (A) light–dark box, where cre^+^ mice also spent more time in the light. **(B)** Elevated plus maze also revealed more visits to the open arms as well as longer distance traveled and increased time spent in the center of the maze. **(C)** Exploratory behavior increased, judged by increased exploration of a novel object. **(D)** In Y-maze, mice did not show a difference in spontaneous alternation. **(E)** Fear conditioning shows decreased contextual as well as cue-dependent learning. Plots depict mean ± SEM, individual data points are provided. Statistics: (A–D) Bayesian analysis, accuracy, * >80%, ** >90%, *** >95% (see Data S1); (E) two-way ANOVA, * P < 0.05, ** P < 0.01.

To examine how IL-4R deficiency in neuronal network activity translates to behavior, we used a battery of tests for anxiety (light–dark box, elevated plus maze) as well as general learning and memory (novel object interaction, Y-maze, Morris water maze, and fear conditioning; Fig. 7 and Data S1). In the light–dark box test, cre^+^ mice spent significantly more time in the light, pointing to lower anxiety levels, and displayed longer walking distances, a typical feature of hyperlocomotion (Fig. 7 A). This behavior was also found in the elevated plus maze, where cre^+^ mice spent more time in the open arms than cre^−^ controls (suggesting lower anxiety levels in cre^+^ mice) and traveled farther (Fig. 7 B). In addition, cre^+^ mice spent more time in the center of the elevated plus maze, which may hint at higher exploratory behavior. We, therefore, analyzed exploratory behavior, finding longer interaction times with a novel object by cre^+^ mice when compared with cre^−^ controls (Fig. 7 C). When testing for general learning and memory, cre^+^ mice were comparable with control cre^−^ mice, as shown by the Y-maze (Fig. 7 D) and Morris water maze (Fig. S2, M–O). The combination of learning and anxiety, however, as assessed by fear conditioning displayed a reduction in the performance of cre^+^ mice; this was observed for the freezing response to both the context as well as to the cue (Fig. 7 E). These data show that mice lacking functional IL-4Rα signaling display a generalized increase in exploratory behavior and locomotion, together with decreased anxiety levels, which were associated with impaired stimulus-dependent fear learning. Interestingly, il4ra^fl/fl^.CamKIIα cre^+^ mice showed normal locomotion in the open field and increased anxiety behavior, apparent as decreased time spent in the center of the open field (Fig. S3 F). Similarly, il4ra^fl/fl^.CamKIIα cre^+^ mice exhibited an increased response to the conditioned cue in fear conditioning (Fig. S3 G). Since these results are in contrast to the behavioral findings in IL-4Rα Syn cre^+^ mice, the opposite phenotype of CamKIIα Cre transgenic mice suggests a strong influence of the inhibitory system in the Syn cre-driven IL-4Rα–deficient line.

Ultimately, neuronal IL-4Rα signaling plays a role in synaptic fine-tuning on both the gene expression and functional level. Pan-neuronal IL-4Rα deficiency leads to abnormal network activity, hyperlocomotion, and higher exploratory behavior, as well as anxiety-related learning deficits, but does not contribute to general learning or memory.

## Discussion

Modulation of neuronal activity by immune signals has mainly been linked to lymphocytes and neuroinflammatory, infectious, or neurodegenerative diseases. However, accumulating evidence indicates that cytokine signaling in the CNS is essential for development and maintenance of homeostatic functions (Salvador et al., 2021). Based on early observations revealing poor cognitive performance in immunodeficient mice, roles of cytokines such as IL-1, IL-6, and IL-1β in memory functions have been suggested (Gadani et al., 2012). Previous studies observed impairment of learning based on the Morris water maze test. IL-4^−/−^ phenotypes could be rescued by IL-4–expressing T cells (Derecki et al., 2010). An IL-4R–dependent pathway was proposed, putatively via astrocytes that produce pro-cognitive brain-derived neurotrophic factor (BDNF) levels after exposure to IL-4. Furthermore, IL-13 may play a role since IL-13^−/−^ mice display similar deficits in learning behavior (Brombacher et al., 2017). We, however, recently provided evidence for the direct signaling of IL-4 on neurons in the context of CNS inflammation (Vogelaar et al., 2018). Intrathecal IL-4 administration ameliorated disease symptoms in a mouse model of autoimmune CNS inflammation via a fast neuronal pathway. It was, however, unclear whether the role of neuronal IL-4R signaling in the CNS was inflammatory-specific and only dependent on exogenous IL-4 treatment, or if neurons also rely on IL-4/IL-4R signaling in homeostatic conditions. Now, we have unraveled a role for IL-4Rα in regulating presynaptic vesicle release via PKC, which is known to regulate synaptic function (Angliker and Ruegg, 2013), resulting in higher neuronal network excitability and eventually in an increased network synchronicity of IL-4Rα–deficient mice. On the behavioral level, this translated into increased exploratory behavior and locomotion, accompanied by decreased anxiety levels and deficits in fear learning. Neuronal IL-4Rα, therefore, plays a distinct modulatory role in cognition, with no obvious effects on basic learning tasks like Y-maze or Morris water maze, but a specific impact on anxiety and fear learning when neuronal IL-4Rα is absent in the CNS. Importantly, we have evidence for the expression of IL-4 in healthy CNS. This is in accordance with previous reports on IL-4 expression by microglia (Ponomarev et al., 2007).

We found presynaptic localization of IL-4Rα association with Bassoon, Synapsin 1, VGLUT1, and VGAT, indicating widespread expression in both excitatory and inhibitory neurons. IL-4Rα was also localized presynaptically in human postmortem brain tissue. ImmunoEM confirmed the presence of IL-4Rα on the presynaptic membrane as well as on synaptic vesicles. The latter finding suggests internalization of the receptor during recycling. Importantly, no differences in dendritic morphology or spine densities were observed, arguing against developmental deficits.

Notably, the expression of different cytokine receptors has also been shown in the peripheral nervous system. IL-4Rα–expressing sensory neurons can be found in dorsal root ganglia. Exposure to IL-4 increases the sensitivity to pruritogens, thereby amplifying chronic itch (Oetjen et al., 2017). These findings underline the common principle that IL-4 has the capacity to act directly on neurons, instead of mediating anti-inflammatory properties on immune, glial, or other bystander cells. As we are just beginning to unravel the pattern of cytokine receptor expression in neuronal subtypes and anatomical areas throughout the CNS, it will be important to consider whether some neuronal circuits are more susceptible to cytokine modulation than others and whether different cytokines have synergistic or antagonistic functions. Importantly, direct IFNγ-signaling in inhibitory neurons in the prefrontal cortex was previously associated with an increase in both neuronal activity and behavioral deficits (Filiano et al., 2016). In the context of experimental autoimmune encephalomyelitis, we recently observed TNFα-induced neuronal hyperactivity during the remission phase, ultimately leading to neuronal death and behavioral abnormalities (Ellwardt et al., 2018). Although studies so far have been performed with different experimental approaches, behavioral disturbances are emerging as leading paradigms of cytokine-mediated homeostatic CNS modulation.

Our unbiased approach investigating the effects of neuronal IL-4Rα deficiency on the transcriptome of adult murine CNS neurons indicated a role for IL-4Rα signaling in the regulation of synaptic gene expression. In fact, GO revealed several factors directly involved in neurotransmission as well as functional synaptic components like Unc13A and C, SNAP-25, Stx1a, Syt7, and Syt12, and structural synaptic components like Bassoon, speaking mainly for a relevant involvement of the presynapse. Indeed, SNAP-25 and Syntaxin are components of the SNARE (soluble *N*-ethylmaleimide-sensitive-factor attachment receptor) complex, with SNAP-25 being essential for evoked synaptic transmission (Washbourne et al., 2002). Synaptotagmin is a Ca^2+^ sensor that regulates vesicle-SNARE interaction; Syt 7 is known to decrease vesicle replenishment rate (Liu et al., 2014), while the Unc13 protein family is involved in SNARE complex assembly and vesicle priming (Dittman, 2019; Imig et al., 2014). Thus, neuronal IL-4Rα deficiency led to the downregulation of genes involved in Ca^2+^-dependent vesicle release, which is in line with our electrophysiological recordings in patched neurons and the reduction of the vesicle pools. Notably, some genes (such as Adgrb1 and Sez6) were regulated in the same direction in cultured IL-4–treated neurons and IL-4Rα–deficient neurons. Although this seems contradictory, the life-long effects of IL-4Rα deficiency in vivo cannot be directly compared with the IL-4 effects in cultured neurons. The alterations we observed in neuronal network activity throughout the IL-4Rα–deficient animals’ lives can themselves lead to changes in gene expression due to signaling from the synapse to the nucleus (Ch’ng and Martin, 2011; Jordan and Kreutz, 2009). A similar and unexpected phenomenon was observed at the level of the patched synapse, where both IL-4Rα–deficient neurons and IL-4–treated neurons displayed a reduction in mEPSC frequencies. Long-term IL-4Rα–deficiency led to a reduction in available pools of synaptic vesicles and the downregulation of various synaptic genes necessary for release, which is not applicable to acute short-term (10 min) effects of IL-4.

Regulators of synaptic vesicle release, such as Synapsin 1 (Cesca et al., 2010; Rosahl et al., 1995) and PKC (Stevens and Sullivan, 1998), also displayed differential expression. It is worth noting that PKCγ is part of our previously identified neuronal IL-4R signaling pathway (Vogelaar et al., 2018), and in our present study underwent phosphorylation in synaptosomes in response to IL-4. Importantly, the upregulation of IPSCs upon acute IL-4 treatment was abolished by inhibition of PKCγ, known to upregulate synaptic transmission (Stevens and Sullivan, 1998).

PKC is particularly interesting because of its known role in upregulating the RRP (Stevens and Sullivan, 1998) and its ability to phosphorylate SNAP-25 (Nagy et al., 2002). A recent study showed that reduced SNAP-25 protein levels affected mEPSC frequency in cultured hippocampal neurons (Fossati et al., 2015). In line with this, evoked potentials via train stimulation revealed reduced exocytosis of the RRP and a reduction in the recruitment of the resting pool of synaptic vesicles of cortical neurons derived from IL-4Rα–deficient mice, implying an inability to refill the RRP. These data were corroborated by our EM findings, showing that IL-4Rα–deficient synapses displayed lower total numbers of synaptic vesicles, and by electrophysiological recordings on the single neuron level, displaying reduced frequencies of synaptic miniature currents. This led to a net increase in spontaneous PSC frequencies, which is consistent with higher neuronal firing resulting from the alteration of both excitatory and inhibitory transmission. Indeed, theoretical synaptic models reported that changes in excitatory synaptic strength affect the threshold of neuronal input–output function, while changes in inhibitory synaptic strength alters the threshold and gain (Carvalho and Buonomano, 2009; Hennequin et al., 2017). In the present case, in the il4ra.Syn cre^+^ mice, where IL-4 signaling is deleted in excitatory and inhibitory synapses (Cesca et al., 2010; Micheva et al., 2010; Zhu et al., 2001), the threshold does not alter due to equalized opposing effects. However, since lower inhibition also alters the gain, the same synaptic activity results in higher neuronal firing. In il4ra.CaMKIIα cre^+^ mice, however, IL-4–deficient signaling in excitatory synapses (Casanova et al., 2001; Wang et al., 2013) alters the threshold toward lower neuronal excitability, as shown by reduced sEPSCs and opposing behavioral changes. On the network level, concomitant changes in excitatory and inhibitory release as observed in the il4ra.Syn cre^+^ mice resulted in an increase in excitatory drive and subsequently in vivo in an increase in power of both θ and γ band oscillations, suggesting a shift of the balance toward network overexcitation. A concurrent publication found that the depletion of CD4 T cells disrupted contextual fear memory and demonstrated inhibitory neurons to be essential for the role of IL-4 for regulating task-activated neurons in the dentate gyrus and for memory deficits (Herz et al., 2021). Our data are further in line with previous findings showing that increased cortical network excitability in the γ range led to an endophenotype of psychiatric disorders showing motor hyperactivity on the behavioral level (Schneider et al., 2018; Thalman et al., 2018). Thus, we can conclude that the impairment of the inhibitory synapses in the Syn cre-driven mice predominantly caused the functional deficits. We here demonstrate that IL-4 is present within the brain parenchyma and that IL-4Rα deficiency leads to reduced anxiety levels with impaired fear stimulus learning and exploratory drive while general learning is not affected. Corroborating this observation, IL-4 treatment of wild-type neurons shifted the balance toward increased frequency of inhibitory currents, suggesting that IL-4 can modulate the balance toward increased inhibition, thereby reducing the risk of hyperexcitation. Finally, IL-4Rα deficiency led to deficits on the synaptic and neuronal network level causing behavioral abnormalities with overall hyperactivity or restlessness.

Our findings on the transcriptional level together with IL-4Rα expression at the presynapse and the connection to presynaptic molecules indicate the involvement of IL-4Rα signaling in synaptic transmission. Phosphorylation of PKC in IL-4–stimulated synaptosomes and abolishment of acute IL-4 effects upon PKC inhibition as well as the availability of IL-4 itself within the CNS point to a novel regulatory mechanism at the presynapse, which fine-tunes the strength of synaptic transmission in the CNS, thus revealing a therapeutic potential at the level of neuronal activity.

## Materials and methods

### Human material

Inferior parietal lobe from a control individual with no history of neurological disease was used for immunological staining (Vogt et al., 2009). The acquisition and handling of human tissue were approved by the Institutional Review Board of the Albert Einstein College of Medicine, protocol #89-31. Human iPSCs were derived from fibroblasts obtained from a healthy subject under informed consent. The study was approved by the local ethical committees of Münster and Milan (AZ 2018-040-f-S and Banca INSpe).

### Animals

C57Bl/6J mice were purchased from Janvier. B6.Cg-Tg(Syn1-cre)671Jxm/J mice (here referred to as Syn cre) and CamKIIα cre mice were provided by Gunther Schütz (Deutsches Krebsforschungszentrum, Heidelberg, Germany); BALB/c-il4ratm1Sz/J (full il4r-deficient mice, here called il4ra^−/−^), B6.129P2-Il4tm1Cgn/J (IL-4^−/−^), and BALB-c/J control mice were purchased from The Jackson Laboratory. Gad67-GFP mice were provided by Thomas Mittmann (University of Mainz, Mainz, Germany). Il4ra.flox mice (here called il4ra^fl/fl^) were originally provided by Frank Brombacher (University of Cape Town, Cape Town, South Africa; Herbert et al., 2004) and backcrossed to C57Bl/6 background (generation F8) by Thomas Kammertöns (Charité, Berlin, Germany). Il4ra^fl/fl^ mice were bred in-house with either Syn cre (Zhu et al., 2001) or CamKIIα cre mice (Casanova et al., 2001) to achieve cell type–specific IL4Rα deficiency in all classically transmitting neuronal cells or in excitatory neurons, respectively. Because of a known possibility of germline recombination in the Syn Cre offspring (Luo et al., 2020), all animals were checked for germline recombination. To this end, in addition to the genotyping protocol for the first LoxP site, we included primers for the detection of the recombined DNA allele as well as copy DNA (cDNA) primers for the detection of the targeted exons in RNA of isolated neurons (Table S1). Only fl/fl mice without germline recombination were used in this study, thus ensuring exclusive neuronal recombination.

All mice were housed in the Translational Animal Research Center of the University Medicine Mainz under a 12/12 h light/dark cycle and had access to water and food ad libitum. Animal procedures were performed in accordance with the European Union normative for care and use of experimental animals, conducted according to the German Animal Protection Law, and approved by the appropriate state committees for animal welfare (Landesuntersuchungsamt Rheinland-Pfalz).

### RNAscope and BaseScope

RNAscope in situ hybridization was performed using the Multiplex Fluorescence v2 (323100; Advanced Cell Diagnostics [ACD]) and 4 Plex Ancillary kit (323120; ACD). Briefly, 30-µm thick sections of adult (10- to 15-wk-old) C57Bl/6J mouse brains were mounted on Superfrost Plus Gold slides (Menzel) and dehydrated in a series of rising ethanol concentrations (50, 70, and 2× 100%). After incubation with hydrogen peroxide for 10 min at room temperature and antigen retrieval in 1× RNAscope target retrieval solution (ACD) for 5 min at 99°C, sections were incubated with Protease III (ACD) for 30 min at 40°C. The following target probes were used in this study (all from ACD): Mm-Il4ra-C1 (520171), Mm-Nefh-C4 (443671), Vglut1: Mm-Slc17a7-C2 (416631-C2), and Vgat: Mm-Slc32a1-C2 (319191-C2). Hybridization was performed for 2 h at 40°C. Subsequent signal amplification and detection was performed according to the manufacturer’s protocol using OPAL 570 (1:1,500), OPAL 520 (1:1,500), or OPAL 690 (1:1,000) tyramide reagents (NEL811001KT; All Akoya Biosciences). After DAPI counterstaining for 30 s, sections were mounted using Pro Long Gold Antifade reagent.

To confirm IL-4Rα deficiency, the deletion of exon 7–9 (Herbert et al., 2004; Noben-Trauth et al., 1997) was analyzed in il4ra^fl/fl^.Syn cre brain sections using the BaseScope Duplex reagent kit (ACD) according to the manufacturer’s instructions. Pre-hybridization steps were the same as for RNAscope. Hybridization with a probe targeting exon 7 of the *il4ra* mRNA (1043121-C1; ACD) and *nefh* mRNA (1090361-C2; ACD) was performed for 2 h at 40°C. Subsequent signal amplification and detection were performed according to the manufacturer’s protocol. Sections were counterstained with 50% Gill’s Hematoxylin solution (Sigma-Aldrich) and mounted in Vectamount mounting medium (H-5000; Vector Laboratories).

### Isolation of neurons from adult murine brains

Whole brains from adult female 10- to 15-wk-old mice were dissociated using the Adult Brain Dissociation Kit and Gentle Macs Octo Dissociator with Heaters (Miltenyi) according to the manufacturer’s protocol. Non-neuronal cells were removed using the Neuron Isolation Kit (Miltenyi), which contains biotinylated antibodies against microglia, oligodendrocytes, and astrocytes, conjugated to magnetic microbeads that are removed by magnetic-activated cell sorting (MACS). The flow-through containing highly enriched neuronal cells was collected. The purity of neuronal isolates was assessed by flow cytometry using a FACS Canto II (BD Biosciences) according to the manufacturer’s instructions. In brief, living cells were gated for single cells only (side scatter and forward scatter), and the samples were stained for cell populations using specific antibodies for microglia/macrophages (CD11b PeCy7; Invitrogen), oligodendrocytes (O4 APC; Miltenyi), endothelial cells (CD31 PEVio770; Miltenyi), thrombocytes/immune cells (CD45 FITC; Biolegend), and astrocytes (astrocyte cell surface antigen-2 [ACSA-2] PE, Miltenyi; Table 1). Neuronal purity of 90–96% was achieved.

**Table 1.** Antibodies used in the study

Technique	Antigen name	Antigen	Host	Company	Catalog number
MACS/FACS	Astrocyte cell surface antigen-2	Acsa-2-Pe	hu	Miltenyi	130-116-244
	CD11b	CD11b-PeCy7	rat	Invitrogen	25-0112-82
	CD31	CD31-Pe-Vio770	ms	Miltenyi	130-119-894
	CD45	CD45-FITC	rat	Biolegend	103107
	Oligodendrocyte marker O4	O4-APC	ms	Miltenyi	130-119-155
Western	β-Actin	Beta actin	ms	Sigma	A5441
	Growth-associated protein-43	GAP-43	rb	Abcam	ab16053
	Protein kinase gamma (phospho T674)	pPKCg	rb	Abcam	Ab5797
	Protein kinase gamma	PKCg	ms	Santa Cruz	sc-166451
	Phospho mitogen-activated protein kinase (phospho extracellular signal-regulated kinase 1/2)	p-P44/42 MAPK (pERK1/2)	rb	Cell Signaling	4370
	Mitogen-activated protein kinase (extracellular signal–regulated kinase 1/2)	P44/42 MAPK (ERK1/2)	rb	Cell Signaling	9,102
IHC/ICC/STED	Bassoon	Bassoon	ms	Synaptic Systems	141021
	Ca^2+^/calmodulin-dependent protein kinase II	CamkIIα	ms	Abcam	Ab22609
	Homer-1	Homer-1	ch	Synaptic Systems	160006
	Interleukin-4 receptor alpha	CD124	rat	BD	551853
	Interleukin-4 receptor alpha	CD124-AF647	rat	BD	564084
	Interleukin-4 receptor alpha	IL-4R	rb	Abcam	ab203398
	Microtubule-associated protein 2	MAP-2	rb	Abcam	ab32454
	Synapsin 1	Syn1	ms	Synaptic Systems	160001
	Vesicular GABA transporter	VGAT	ms	Synaptic Systems	131011
	Vesicular glutamate transporter 1	VGLUT1	ms	Synaptic Systems	135011

ICC, immunocytochemistry; ch, chicken; hu, human; ms, mouse; rb, rabbit.

### Human neurons from iPSCs

Fibroblasts were obtained under informed consent from a healthy control subject. They were reprogrammed into iPSCs using the replication incompetent Sendai reprogramming kit (Invitrogen) according to the manufacturer’s instructions. Human iPSC–derived NPCs were expanded, and differentiation of NPCs was performed as previously described (Reinhardt et al., 2013). Briefly, NPCs were cultured on matrigel-coated (Corning) 12-well plates (Nunc; Thermo Fisher Scientific) in N2B27 medium (DMEM-F12 [Gibco] and neurobasal medium [Gibco; 1:1], 1% B27 without vitamin A [Gibco], 0.5% N2 [Gibco], penicillin/streptomycin/L-glutamine [Gibco]), supplemented with 3 µM CHIR99021 (Axon Mechem), 1 µM smoothened agonist (Cayman Chemical) and 200 µM ascorbic acid (AA; Sigma-Aldrich). To initiate neuronal differentiation, NPCs were cultured in N2B27 medium supplemented with 1 µM smoothened agonist, 10 ng/ml BDNF (Peprotech), 10 ng/ml glial cell–derived neurotrophic factor (Peprotech), and 100 µM AA for 6 d followed by N2B27 medium supplemented with 10 ng/ml BDNF, 10 ng/ml glial cell–derived neurotrophic factor, 1 ng/ml TGF-β3 (Peprotech), 0.5 mM dibutyryl-cAMP (Sigma-Aldrich), and 100 µM AA. Additionally, 5 ng/ml Activin A (Peprotech) was added to the medium from day 7 to day 9. Neuronal cultures were split with Accutase (Gibco) between day 14 and day 16 after initiation of neuronal differentiation and reseeded on fresh matrigel-coated culture plates until analysis. At 35 d after differentiation, neurons were treated with 50 ng/ml human recombinant IL-4 (Peprotech) for 7 d.

### RNA isolation and bulk RNAseq

RNA was isolated from murine and human neurons using the RNeasy Micro Kit (Qiagen) according to the manufacturer’s instructions. The quantity of total RNA was measured using a Qubit 2.0 Fluorimeter (Invitrogen) and the quality was assessed using a RNA 6000 Nano chip on a Bioanalyzer 2100 (Agilent Genomics). Only samples with RNA integrity numbers ≥7 were included in library preparation. Sequencing libraries were prepared from 50 ng (human) and 10 ng (murine) total RNA using the NEBNext Ultra II RNA Library Prep Kit for Illumina (New England Biolabs), according to the manufacturer’s protocol for polyA mRNA workflow. Quality of the cDNA library preparation was checked using the Agilent High Sensitivity DNA assay (Agilent) on a Bioanalyzer 2100 platform. The resulting barcoded cDNA libraries were sequenced on an Illumina NovaSeq6000 (Novogene) with a S4 FlowCell with PE150 bp (300 cycles).

Raw RNAseq data were filtered with the fastp program (Chen et al., 2018) for eliminating low-quality reads. Parameters -g -x -p were set for polyG tail trimming, polyX tail trimming, and overrepresented sequence analysis. The quality of trimmed data was verified with the fastqc program (Babraham Bioinformatics). Afterward, data were aligned to the latest reference genome (GRCm39 for the mouse and GRCh38.p13 for the human) using the long-read STAR aligner (Dobin et al., 2013). Low-quality alignments were filtered out using SAMtools (Li et al., 2009). The remaining high-quality alignments were quantified using StringTie (Pertea et al., 2015). Statistical analysis of the gene counts was carried out with DESeq2 (Love et al., 2014) in R. GO analysis was performed with clusterProfiler and DOSE and visualized with Enrichplot (Yu et al., 2015). The databases org.Hs.eg.db and org.Mm.eg.db were used in R for annotating purposes; heatmaps were created with pheatmap package in R.

Raw data from mouse bulk mRNA sequencing of the present study have been deposited in the National Center for Biotechnology Information’s Gene Expression Omnibus (Edgar et al., 2002) and is accessible via the accession code GSE200569. Raw data from human single-cell RNAseq of the present study have been deposited in the National Center for Biotechnology Information’s Gene Expression Omnibus under the accession code GSE200555.

### Reverse transcriptase quantitative PCR (RT-qPCR)

Murine dissociated neurons were prepared as previously described (Walsh et al., 2015). After 5 d in vitro, neurons were treated with 50 ng/ml murine recombinant IL-4 (Peprotech) or PBS for 7 d. Cells were homogenized in lysis buffer, and RNA was isolated using RNeasy micro kit (Qiagen) according to the manufacturer’s instructions. RT-qPCR was performed as previously described (Jolivel et al., 2013). Primers are listed in Table 2. Ribosomal protein S29 (RPS29) was used as a housekeeping gene. For RT-qPCR on tissue, mice were perfused with PBS and their brains were removed for dissection of the cortex and hippocampus. Spleens were removed shortly before perfusion. Tissue was processed to RNA and qPCR for IL-4 (Table 2) and RPS29 was performed.

**Table 2.** Primers used in the study

Gene	Forward primer (5′–3′)	Reverse primer (5′–3′)
*Adhesion G protein–coupled receptor B1*	CTGAGAAGCAAACCAAGT	GACCATTCGTTCCAGTTT
*Interleukin-4*	TCA​TCC​TGC​TCT​TCT​TTC​TC	TCCTGTGACCTCGTTCAA
*Netrin 1*	CCCTTCCAGTTCTATTCC	TCGTTCTGTTTGGTGATA
*Ribosomal protein S29*	CAAATACGGGCTGAACAT	GTC​GCT​TAG​TCC​AAC​TTA​A
*Seizure-related 6 homolog*	CACCATCATTACCACTAC	GTA​GAC​AGA​GAT​ATA​GTA​GAA

### IHC of murine brain

Vibratome (HM650V; Thermo Fisher Scientific) sections of C57Bl/6 or Gad67-GFP mouse brains (the latter is a reporter line for inhibitory neurons) were stained using the following primary antibodies: IL-4Rα-Alexa 647 (BD Bioscience) and CamkIIα (Abcam; Table 1). Alexa-fluorophore–coupled secondary antibodies (Merck Millipore) and DAPI were used for visualization.

### Immunocytochemistry of human neurons

Neuron cultures (35–42 d after differentiation) on glass coverslips were fixed with 4% paraformaldehyde (PFA) and washed with PBS. After blocking and permeabilization with 5% normal goat serum (Vector), 1% BSA (Sigma-Aldrich) and 0.2% Triton- X-100 for 1 h at room temperature, cells were incubated with primary antibodies for mouse-anti-Syn1 (Synaptic Systems), chicken-anti-Homer-1 (Synaptic Systems), and rabbit-anti-MAP-2 (Abcam; Table 1) overnight at 4°C. After washing, secondary labeling was performed using appropriate Alexa Fluor-conjugated goat secondary antibodies (Invitrogen) for 2 h at room temperature. Coverslips were mounted using Vectamount AQ (H-5501; Vector Laboratories).

### Stimulated emission depletion microscopy (STED)

For STED microscopy, 50 µm thick vibratome (HM650V; Thermo Fisher Scientific) brain sections were blocked and permeabilized with 10% normal goat serum and 0.2% Triton X-100 in PBS for 1 h at room temperature. Primary antibody incubation was performed sequentially at 4°C for 48 h using the following antibodies: rabbit-anti-IL-4Rα (Abcam), mouse-anti-Syn1 (Synaptic Systems), mouse-anti-Bassoon (Synaptic Systems), mouse-anti-VGAT (Synaptic Systems), VGLUT1 (Synaptic Systems), and chicken-anti-Homer-1 (Synaptic Systems). Secondary labeling was performed for 24 h at 4°C with goat-anti-rabbit STAR 580 (Abberior), goat-anti-mouse STAR 635P (Abberior), and goat-anti-chicken Alexa Fluor 488 (1:500, A-11039; Invitrogen). Sections were mounted with Pro Long Gold Antifade reagent and imaged on a Leica TCS SP8 STED 3× equipped with a white light laser and 592 and 775 nm depletion lasers. τ-STED microscopy was performed using a HC PL APO CS2 93×/1.30 Glyc objective. For the quantification of colocalization of IL-4Rα with excitatory and inhibitory synapses in hippocampal stratum radiatum, signals were processed separately in each channel. Pixel intensities were normalized to 0–255 range, and high-intensity regions were identified by iteratively finding regions above a given cut-off with a size >25 pixels (∼0.497 µm). The intensity the cut off was decreased in each iteration, from 255 to 95, a value chosen based on visual inspection of correlative stained images (with IL-4Rα/VGLUT1 or IL-4Rα/VGAT) and visibility of pixel regions (as applied by Greenwald et al. [2021]). Each pixel was labeled as a connected component (Samet, 1988) and region center points were determined based on their moments (Hu, 1962). Round regions with a radius *r* = 4 pixels (∼0.080 µm) were considered to represent synapses (Bourne and Harris, 2008). Overlaps of round regions with radius *r* = 4 between channels were considered colocalizations. To prevent overestimation, margins of *r* = 10 for VGAT and *r* = 5 for VGLUT1 (due to higher density) were set around each synapse to exclude further high-intensity region identification.

### EM

EM was performed using standard procedures as previously described (Trimbuch et al., 2009). Briefly, following animal perfusion with 4% PFA, 0.1% glutaraldehyde, and 15% picric acid in 0.1 M phosphate buffer (pH 7.4), brains were isolated, postfixed, and cut on a vibratome in 50-µm thick slices. Following a freeze–thaw protocol, sections were incubated with rabbit-anti-IL-4Rα (Abcam) for 96 h at 4°C and subsequently with goat-anti-rabbit secondary antibody conjugated to 1.4 nm Nanogold (Nanoprobes) for 96 h at 4°C, followed by embedding in EPON 812 resin. Ultrathin slices (60 nm) of the stratum radiatum of the hippocampal CA1-region were cut on a Leica UCT ultramicrotome and imaged on a transmission electron microscope Zeiss 912. For assessment of presynaptic vesicles, animals were perfused with 2% PFA, 2.5% glutaraldehyde, and 15% picric acid in 0.1 M phosphate buffer (pH 7.4) and processed for fine structural analysis as described above. Quantitative analysis of presynaptic vesicle distribution was performed in 50 nm bins from the synaptic cleft. A total of 72–73 synapses were analyzed for each genotype (*n* = 6 cre^+^ animals and *n* = 6 cre^−^ animals).

### Ultrapure synaptosomes isolation

Synaptosome isolation was performed according to Dunkley et al. (2008). Briefly, the brains from 10- to 15-wk-old mice (male and female, mixed) were homogenized in buffer (0.32 M sucrose, 1 mM EDTA, 5 mM Tris, pH 7.4) and centrifuged at 950 *g* for 10 min at 4°C. Supernatants (S1) were collected. Pellets were resuspended in 10 ml homogenization buffer, centrifuged at 950 *g* for 10 min at 4°C, and these S2 supernatants were combined with S1. After the addition of 0.25 mM dithiothreitol, the suspension was layered over a 4 × 5 ml Percoll gradient (23, 15, 10, 3% Percoll [v/v], pH 7.4) and centrifuged at 31,400 *g* (SW28 rotor) for 5 min at 4°C with slow acceleration/deceleration in the Beckman Coulter OptimaTM L-80 XP ultracentrifuge. The F4 fraction was harvested and transferred into ice-cold homogenization buffer, followed by centrifugation for 30 min at 31,400 *g* at 4°C. Pellets were washed with ice-cold Krebs buffer (118 mM NaCl, 5 mM KCl, 25 mM NaHCO_3_, 1 mM MgCl_2_, 10 mM glucose) and resuspended in Krebs buffer containing 2 mM Ca^2+^. These ultrapure synaptosomes were then incubated with 50 ng/ml of recombinant murine IL-4 (Peprotech) or equal volumes of PBS for 10 min at 37°C. Phosphatase and protease inhibitors (50 mM sodium fluoride, 1 mM sodium orthovanadate, 2.5 mM sodium pyrophosphate, 1 mM sodium β glycerophosphate and 1× cOmplete, EDTA-free Protease Inhibitor 50× [ROCHE-51765505]) were used in all solutions.

### Western blotting

For Western blotting, anti-phospho-PKCγ (Abcam) and anti–β-actin (loading control; Sigma-Aldrich) were used to detect IL-4 effects on synaptosomes. Anti-P44/42 MAPK (ERK1/2) and anti–phospho-P44/42 MAPK (ERK1/2) were used on Western blots of IL-4–stimulated dissociated neurons (50 ng/ml, 10 min). DyLight 800/600-coupled secondary antibodies were used for quantitative analysis of the proteins using the Li-Cor Odyssey FC imaging system (Li-Cor Bioscience) and ImageJ. For co-IP, performed as previously described (Vogelaar et al., 2018), synaptosome lysates were incubated with anti–IL-4α (BD Bioscience) on protein-A/G agarose beads overnight at 4°C in IP buffer (20 mM Tris-HCL pH 7.5, 150 mM NaCl, 5 mM EDTA, 1 µg/ml pepstatin; Sigma-Aldrich). Precipitation was performed by centrifugation with descending speeds (6,000–3,000 *g*) for 2 min. Blots were incubated with anti–β-actin (Sigma-Aldrich), anti-PKCγ (Santa Cruz), and anti–GAP-43 (Novus Biologicals; Table 1). Raw data were extracted from the Li-Cor software and imported to ImageJ using the bio-formats plugin. Bands were outlined with the rectangular icon and intensities were plotted in curves. The area under the curve was calculated for each sample.

### Electrophysiological recordings

Experiments were performed as previously described (Thalman et al., 2018) on CA1 neurons in acute 350 µm thick horizontal brain slices from 29- to 35-d-old male and female (mixed) il4ra^fl/fl^.Syn cre^+^ and cre^−^ littermates or C57Bl/6J controls. Briefly, whole-cell patch-clamp recordings in hippocampal CA1 were performed in artificial cerebrospinal fluid composed of (in mM) 126 NaCl, 2.5 KCl, 10 glucose, 1.25 NaH_2_PO_4_, 26 NaHCO_3_, 2 CaCl_2_, and 1 MgCl_2_. Spontaneous and miniature excitatory and inhibitory postsynaptic currents (sPSCs, mEPSCs, mIPSCs) were acquired with an ELC-03XS amplifier (NPI Electronics) and Spike2 software (Cambridge Electronic Design products). Signals were filtered at 2 kHz and sampled at a rate of 10 kHz. Recordings were performed with a GB200F-10 filament microelectrode (3–7 MΩ) in CA1 neurons held at −70 mV (voltage-clamp). For sPSCs and mEPSCs, microelectrodes were loaded with an intracellular solution containing (in mM): 107 K-Gluconate, 20 KCl, 5 NaCl, 5 ethylene glycol bis (2-aminoethyl ether) tetraacetic acid (EGTA), 20 K-Hepes, 2 Mg-ATP, 0.3 Na-GTP, and 0.5 CaCl_2_. mEPSCs were pharmacologically isolated using tetrodotoxin (0.5 μM; blocking voltage-gated sensitive Na^+^ channels) and gabazine (10 μM; GABA receptor inhibitor). For inhibitory recordings, microelectrodes were loaded with an intracellular solution containing (in mM): 150 KCl, 5 NaCl, 5 EGTA, 20 Hepes, 2 Mg-ATP, 0.3 Na-GTP, and 0.5 CaCl_2_. All solutions were pH 7.2 with osmolarity 310–320 mOsm. mIPSCs were pharmacologically isolated using tetrodotoxin (0.5 μM), 6.7-dinitroquinoxaline-2,3-dione (10 μM), and DL-2-amino-5-phosphonopentanoic acid (50 μM; NMDA-receptor inhibitor). mEPSCs and mIPSCs were analyzed with a custom-made fully automated script for the Spike2 software. Recordings on human neurons were performed under the same conditions. For recording in response to IL-4, after baseline recording, C56BL/6 slices were incubated with 50 ng/ml IL-4 for 10 min. Incubation with the PKC inhibitor BisI (100 nM; Cell Signaling) was performed to investigate dependency on PKC.

For train stimulations, whole-cell voltage-clamp recordings on CA1 neurons were performed in Ringer’s solution at 32°C using an EPC10 double USB patch clamp amplifier (HEKA Elektronic). Ringer’s solution was supplemented with 10 µM gabazine and 50 µM DL-2-amino-5-phosphonopentanoic acid to block GABAergic- and NMDA receptor-mediated currents. Recordings were performed with pulled borosilicate glass (2 mm OD, Hilgenberg; 3–6 MΩ) electrodes, filled with intracellular recording solution containing in mM: 35 Cs-gluconate, 100 CsCl, 10 EGTA, 10 HEPES, and 0.1 D-600; holding potential of CA1 neurons was −70 mV. Stimulation of Schaffer collateral axons was performed using a monopolar borosilicate glass electrode and a broken tip filled with Ringer’s solution, placed in the stratum radiatum. Using a stimulus isolator unit (A365; WPI), 0.1–0.4 mA currents were applied, achieving maximal current responses without eliciting action potentials. Using Patchmaster software, 100 pulses at 20 Hz for 0.1 ms were triggered by the amplifier and current responses were recorded. Stimulation was repeated 10–20 times, current responses averaged, and amplitudes determined using IGOR Pro software’s Neuromatic extension (Rothman and Silver, 2018) and custom code written by Dr. Eric Jacobi (Institute for Pathophysiology, University Medical Center Mainz). Sweeps with action potentials were manually removed during analysis and only cells with ≥10 recorded sweeps were analyzed. Series resistance was continuously monitored by applying a +5 mV pulse at the start of each sweep and cells exceeding 30 MΩ series resistance were omitted. The effective RRP was determined using a modified back extrapolation method (Schneggenburger et al., 1999; Thanawala and Regehr, 2013). Briefly, a cumulative plot of the EPSC amplitudes was established and stimuli numbers 90–100 were used to generate a linear fit. The y-intercept of this fit is a measure of the effective pool estimate. The slope of the fit shows the recruitment rate of synaptic vesicles.

### Sholl and spine analysis

For reconstruction of neuronal morphology and spine analysis, patched neurons were filled with biocytin (Sigma-Aldrich) and fixed with 4% PFA. After washing three times with PBS permeabilization, blocking was performed for 1 h using 5% BSA/PBS supplemented with 0.1% Triton X-100. Labeled cells were visualized with Alexa-488–conjugated streptavidin and coverslipped with ProLong antifade mountant (Thermo Fisher Scientific).

For Sholl analysis, confocal mosaic scans of the complete neuron were captured using the Leica SP5 confocal laser-scanning microscope. Analysis was limited to a 720-μm radius, and any two neurons with massively overlapping dendritic branches were excluded from the analysis. Soma and dendrites were traced with the help of the NeuronJ plugin on ImageJ (National Institutes of Health), and traces were converted to Shock Wave Component (swc) files by the programs Bonfire (Langhammer et al., 2010) and MATLAB (MathWorks). The program NeuronStudio (Icahn School of Medicine at Mount Sinai; Kutzing et al., 2010) was then used to generate the pattern of connections between neurite segments. After checking for errors and non-links, Sholl analysis was performed by drawing concentric circles around the soma with a growing radii of 6 μm, and the number of times each circle was crossed by a dendritic segment was calculated using Bonfire.

For spine analysis, individual high-resolution images of second and third-order apical dendrites were created with the Leica SP5 confocal laser-scanning microscope (63× oil immersion objective [N.A – 1.4] with voxel size 0.048 × 0.048 × 0.503 µm). Deconvolution was performed using the Maximum Likelihood Estimation algorithm with Huygens (Scientific Volume Imaging). Dendritic spine analysis was performed using 64× Imaris 9.2.0 Software (Bitplane), and spines were classified into stubby, mushroom, long/thin, and filopodia with the help of Classify Spines XT file using the following empirically determined parameters: minimum dendrite end diameter 0.2 μm, minimum spine end diameter 0.2 μm, and maximum spine length 4.0 μm. At least 1,000 spines or at least 1,000 µm dendritic length were measured. Spine density was defined as the number of spines per µm dendrite.

### Local field potentials

In vivo electrophysiological recordings were performed in the hippocampus and layer 2/3 of the medial prefrontal cortex (mPFC). Briefly, anesthetized male animals were implanted with a 16-channel probe (Neuronexus Probe A1x16-3mm-50-177) in the mPFC using stereotaxic coordinates (hippocampus: from Bregma 1 mm lateral, −1.70 mm anterior–posterior, 2.5 mm dorsal–ventral; mPFC: from Bregma +0.25 mm lateral, 1.94 mm anterior-posterior and 3.2 mm dorsal-ventral). Recordings were acquired using a micro3 1401 ADC (Cambridge Electronic Design) and Spike2 software (Cambridge Electronic Design) and analyzed using the FieldTrip toolbox (Oostenveld et al., 2011) and custom programs written in MATLAB (version 2019b; MathWorks) as previously published (Koirala et al., 2020; Muthuraman et al., 2020). Briefly, the full sampling rate of 20 kHz was used to analyze local field potential (LFP), spike detection, sorting, and single unit (SU) analysis. Spectral power was split into five different frequency bands (δ = 1–3 Hz, θ = 4–10 Hz, α = 11–15 Hz, β = 16–30 Hz, low γ = 31–70 Hz, and high γ = 71–100 Hz). LFP power was calculated using multitaper frequency transformation. First, the recorded signals were high-pass filtered at 0.1 Hz and low-pass filtered at 250 Hz. The resulting spectra were sorted according to the layer of recording, pooled for all animals, and separated into different frequency bands. Spike detection and sorting were performed using WaveClus 3 (Luan et al., 2018). Recorded wide-band signals were high-pass filtered (0.3–3 kHz) and spike detection was performed in each channel independently using amplitude thresholding in the negative range (threshold level at 5× SD of signal). Peaks that exceeded 30 SD of noise level were excluded. Amplitude values crossing the threshold in the time range from −0.5 to +3 ms relative to the negative peak were extracted using a selection of wavelet coefficients chosen with a Kolmogorov–Smirnov test of normality (Quiroga et al., 2004). Finally, the quality of the SU was analyzed using the MLIB toolbox (Stüttgen, 2021). Incompletely separated or unstable SU were excluded from the analysis. Statistical analysis was performed in MATLAB (version R2019a; MathWorks). Two-way ANOVA with factors as groups and frequency bands were used to identify significant effects. All post-hoc tests were Bonferroni corrected to account for multiple comparisons (e.g., different layer combinations or different frequency bands).

### Behavior

Prior to experiments, adult 9- to 19-wk-old il4ra.Syn cre^+^ and cre^−^ mice were habituated to the experimenter for 5–15 min daily for 5 d. Experiments were recorded by video tracking (Noldus Ethovision). Experiments were performed in the Mouse Behavior Unit of the Focus Program Translational Neurosciences of the Johannes Gutenberg University, Mainz. Animal groups were blinded to the observer throughout the experiment. All mice were subjected to the following tests on the indicated days, with sufficient time between tests to recover from task-related stress:

Open field (day 1): Mice were placed in the center of an open field arena (40 × 40 × 40 cm, length × width × height) and allowed to explore freely for 10 min. Total distance traveled and time spent in the center (defined as a square of 20 × 20 cm) were analyzed.

Elevated plus maze (day 2–3): Mice were placed in a plus-shaped elevated maze (40 cm from floor) with two oppositely positioned open arms perpendicular to two oppositely positioned closed arms (30 × 5 cm, length × width, connected by a central platform 5 cm × 5 cm; walls of closed arms 15 cm high). Mice were placed in the central zone and were allowed to move freely to explore the arms for 5 min under video recording. Total distance traveled, numbers of entries, and time spent in the open arms, omitting the central zone, were determined.

Light–dark box (day 4): Mice were placed in a rectangular box (50 cm × 50 × 50 cm, length × width × height) divided into a light (white walls, illuminated by 100 W light source) and a dark (black walls) compartment, connected by a small opening. Behavior was recorded for 10 min. The number of entries and the time spent in each compartment were analyzed as an indicator of anxiety and the willingness to take risks.

Y-maze (day 8): Mice were placed in the center of a Y-shaped maze composed of three identical arms (30 cm long, 120° apart, dark walls) with video tracking. At the end of each arm, a visual cue was placed to help mice remember into which arm they entered. Mice were free to explore the arms of the maze for 10 min. The number of spontaneous alterations, defined as consecutive entries in three different arms, was assessed.

Morris water maze (day 12–22): Mice were placed in a circular tank (120 cm diameter, 40 cm high) filled with tepid water (25 ± 1°C). By placing cues on the walls to allow the mice to orientate, four quadrants were defined. During the first 2 d, mice were trained to escape via a visual platform (10 cm diameter). On the following 8 d, the platform was placed on the opposite side and hidden 1 cm below the water surface. The latency to find the platform (four trials of 90 s) was measured. On the last day, a probe session (90 s) was performed in which the platform was removed. Mice were placed in the opposite sector of the target zone and time spent in each quadrant was recorded.

Novel object interaction (day 26–27): Mice were placed in the open field, and after 10 min habituation, an object was introduced to the open field. Interaction with the object was recorded for 5 min.

Fear conditioning (day 36–37): This test was carried out in a sound-attenuating box. During the training phase, each mouse was placed in the test cage (20 × 20 × 36 cm, context A). After 2 min of habituation, a conditioning tone (10 kHz, 75 dB, 30 s) sounded, followed by a foot shock (0.4 mA) lasting for 2 s, delivered via a grid floor. This procedure was repeated two times with an inter-trial interval of 20 s. After training, mice were placed back in their home cage. After 24 h of training (on day 37), mice were placed again in context A, and their freezing time was assessed for 2 min. On the same day, mice were habituated to a new box (cylinder, context B) for 2 min and were then exposed again to the conditioning tone. The time that mice were immobile was assessed as a parameter of fear learning.

Data acquisition and analysis: Animal groups were blinded to the observer; video images were recorded and analyzed automatically by Noldus Ethovision software.

### Statistical analysis

Statistical analyses were performed with GraphPad Prism software (version 7) or with the BEST R package (https://jkkweb.sitehost.iu.edu/BEST/) for estimation of the Bayesian posterior distribution. Appropriate statistical tests were chosen based on the experimental condition. Following outlier identification (using ROUT or Grubbs analysis), normal distribution of data was assessed using a corresponding normality test. For experiments involving several potential sources of variance in the datasets (e.g., fear conditioning, Morris water maze), two-way ANOVA was applied. Paired *t* tests were used in experiments where groups were dependent (electrophysiology on the same neuron). For data where two independent groups are compared and for data with a non-Gaussian distribution or data where the animal number and effect size resulted in low power, we used Bayesian posterior distribution analyses to identify differences between the two groups. Since outliers are candidates for aberrant data that may otherwise adversely lead to incorrect results (Liu et al., 2004), to obtain coherent analysis, outlier analysis was performed when necessary. Hereby, we used the univariate method for outlier detection in which an outlier region was defined based on the confidence coefficient α (95% confidence interval or highest density interval). α was defined based on the Monte Carlo simulations for different sample sizes (Davies and Gather, 1993). For these analyses, we used the BEST R package for the Bayesian a priori analyses. Bayesian posterior analysis was performed using the Markov Chain Monte Carlo approach for the choice of priors; the default Markov Chain Monte Carlo sample size of 100,000 was used for all analyses. Bayesian analysis provides complete distributions of credible values for group means and their differences (Kruschke, 2013). This type of analysis for classification and effect size estimation was applied as previously reported (Bahr et al., 2021; Michels et al., 2017). Group differences were calculated as differences in the means and are shown as differences between the groups, which describes the ability to separate the compared groups. Differences with an accuracy ≥80% were interpreted as significant (*). Accuracies ≥90% were regarded as highly significant (**), while differences above 95% between the group values were assigned the highest significance (***). An 80% accuracy for differentiating between two groups is defined by a 20% probability of rejecting a true null hypothesis. This corresponds to a P value of P = 0.05 (Johnson, 2013), hence our cut-off. Reporting guidelines written by the developer of the Bayesian toolbox also confirm that 80% is within the range to accept the null hypothesis with a credible effect size analyzed by Bayesian posterior probability (Kruschke, 2021). In addition, the effect size was calculated and shown for each comparison.

### Online supplemental material

Fig. S1 shows neuronal expression of IL-4Rα. Fig. S2 supports that neuronal IL-4R deficiency has no effect on dendrite morphology, PSC amplitudes and Morris water maze performance. Fig. S3 provides data indicating that lL-4ra^fl/fl^.CamKIIα cre mice show mild phenotypes. Data S1 depicts Bayesian analyses of quantitative experimental data. Table S1 provides an overview of the PCRs used to identify genotypes and germline recombination.

## Supplementary Material

Table S1 lists PCRs for identifying genotypes and germline recombination.

Data S1 contains Bayesian analyses of quantitative experimental data. Bayesian statistics of the posterior predictive distribution (PDD) for quantitative parameters are organized chronologically for Figs. 2, 3, 4, 5, 6, 7, S1, S2, and S3. PDD graphs for difference of means, difference of SD and effect size for the comparisons between two groups. The 95% highest density interval is shown as black lines. Significant differences of difference of means and effect size between groups are defined as accuracy * >80%, ** >90%, *** >95%.

SourceData F2 contains original blots for Fig. 2.

SourceData F4 contains original blots for for Fig. 4.
