# Supplementary material for: Interleukin-4 receptor signaling modulates neuronal network activity
Source: J Exp Med. 2022 May 19;219(6):e20211887. doi: 10.1084/jem.20211887 (PMC9123307; doi:10.1084/jem.20211887)
Supplement: Table S1 — lists PCRs for identifying genotypes and germline recombination. [file JEM_20211887_TableS1.docx]

**Table S1. PCRs for identifying genotypes and germline recombination.**

| **PCR** | **Forward (5′ – 3′)** | **Reverse (5′ – 3′)** |
| --- | --- | --- |
| Flox | CCCTTCCTGGCCCTGAATTT^1^ | GTTTCCTCCTACCGCTGATT^a^ |
| Recombined allele |  | CAGGGAACAGCCCAGAAAAG |
| Ex 3-11 | CCTCTGTGGGCTGTCTGATT | CTTGGTTGACTCCTGGCTTC |

Primers for detection of recombination on DNA and RNA templates. Flox PCR is used for genotyping, recombined PCR is used to detect the recombined allele. RT-PCR for exon 3-11 is used to confirm excision of exon 7-9 at the mRNA level.

^a^from Herbert et al. (2004).

**References**

Herbert, D.R., C. Holscher, M. Mohrs, B. Arendse, A. Schwegmann, M. Radwanska, M. Leeto, R. Kirsch, P. Hall, H. Mossmann, et al. 2004. Alternative macrophage activation is essential for survival during schistosomiasis and downmodulates T helper 1 responses and immunopathology. *Immunity*. 20:623–635. 10.1016/s1074-7613(04)00107-4
