# Supplementary material for: Interleukin-4 receptor signaling modulates neuronal network activity
Source: J Exp Med. 2022 May 19;219(6):e20211887. doi: 10.1084/jem.20211887 (PMC9123307; doi:10.1084/jem.20211887)
Supplement: Data S1 — contains Bayesian analyses of quantitative experimental data. Bayesian statistics of the posterior predictive distribution (PDD) for quantitative parameters are organized chronologically for Figs. 2, 3, 4, 5, 6, 7, S1, S2, and S3. PDD graphs for difference of means, difference of SD and effect size for the comparisons between two groups. The 95% highest density interval is shown as black lines. Significant differences of difference of means and effect size between groups are defined as accuracy * >80%, ** >90%, *** >95%. [file JEM_20211887_DataS1.pdf]

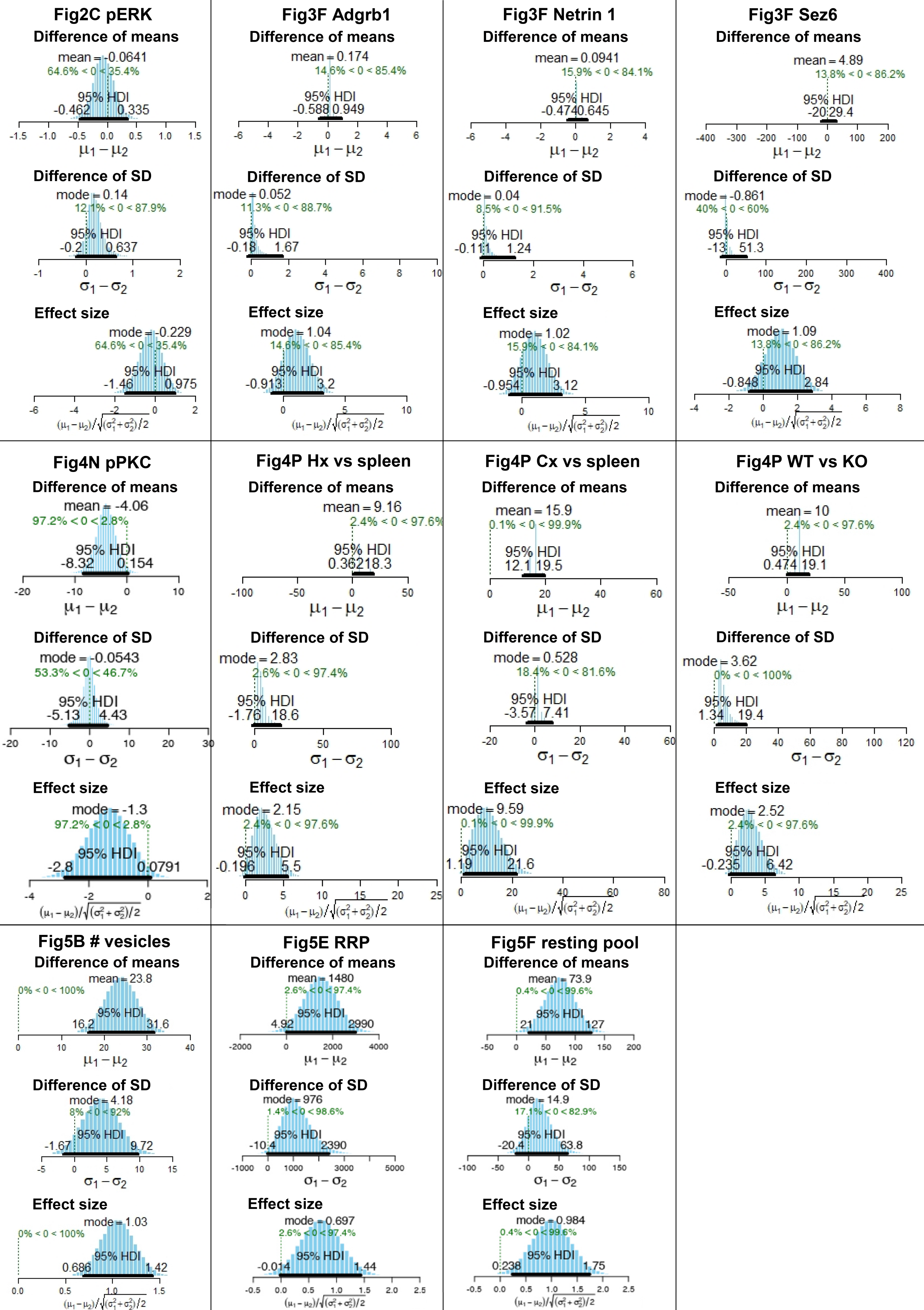
Fig2C pERK
Difference of means
mean = -0.0641
64.6% < 0 < 35.4%
95% HDI
-0.462 0.335
μ1 − μ2
Difference of SD
mode = 0.14
12.1% < 0 < 87.9%
95% HDI
-0.2 0.637
σ1 − σ2
Effect size
mode = -0.229
64.6% < 0 < 35.4%
95% HDI
-1.46 0.975
(μ1−μ2)/√((σ1²+σ2²)/2)
Fig3F Adgrb1
Difference of means
mean = 0.174
14.6% < 0 < 85.4%
95% HDI
-0.588 0.949
μ1 − μ2
Difference of SD
mode = 0.052
11.3% < 0 < 88.7%
95% HDI
-0.18 1.67
σ1 − σ2
Effect size
mode = 1.04
14.6% < 0 < 85.4%
95% HDI
-0.913 3.2
(μ1−μ2)/√((σ1²+σ2²)/2)
Fig3F Netrin 1
Difference of means
mean = 0.0941
15.9% < 0 < 84.1%
95% HDI
-0.474 0.645
μ1 − μ2
Difference of SD
mode = 0.04
8.5% < 0 < 91.5%
95% HDI
-0.111 1.24
σ1 − σ2
Effect size
mode = 1.02
15.9% < 0 < 84.1%
95% HDI
-0.954 3.12
(μ1−μ2)/√((σ1²+σ2²)/2)
Fig3F Sez6
Difference of means
mean = 4.89
13.8% < 0 < 86.2%
95% HDI
-20 29.4
μ1 − μ2
Difference of SD
mode = -0.861
40% < 0 < 60%
95% HDI
-13 51.3
σ1 − σ2
Effect size
mode = 1.09
13.8% < 0 < 86.2%
95% HDI
-0.848 2.84
(μ1−μ2)/√((σ1²+σ2²)/2)
Fig4N pPKC
Difference of means
mean = -4.06
97.2% < 0 < 2.8%
95% HDI
-8.32 0.154
μ1 − μ2
Difference of SD
mode = -0.0543
53.3% < 0 < 46.7%
95% HDI
-5.13 4.43
σ1 − σ2
Effect size
mode = -1.3
97.2% < 0 < 2.8%
95% HDI
-2.8 0.0791
(μ1−μ2)/√((σ1²+σ2²)/2)
Fig4P Hx vs spleen
Difference of means
mean = 9.16
2.4% < 0 < 97.6%
95% HDI
0.362 18.3
μ1 − μ2
Difference of SD
mode = 2.83
2.6% < 0 < 97.4%
95% HDI
-1.76 18.6
σ1 − σ2
Effect size
mode = 2.15
2.4% < 0 < 97.6%
95% HDI
-0.196 5.5
(μ1−μ2)/√((σ1²+σ2²)/2)
Fig4P Cx vs spleen
Difference of means
mean = 15.9
0.1% < 0 < 99.9%
95% HDI
12.1 19.5
μ1 − μ2
Difference of SD
mode = 0.528
18.4% < 0 < 81.6%
95% HDI
-3.57 7.41
σ1 − σ2
Effect size
mode = 9.59
0.1% < 0 < 99.9%
95% HDI
1.19 21.6
(μ1−μ2)/√((σ1²+σ2²)/2)
Fig4P WT vs KO
Difference of means
mean = 10
2.4% < 0 < 97.6%
95% HDI
0.474 19.1
μ1 − μ2
Difference of SD
mode = 3.62
0% < 0 < 100%
95% HDI
1.34 19.4
σ1 − σ2
Effect size
mode = 2.52
2.4% < 0 < 97.6%
95% HDI
-0.235 6.42
(μ1−μ2)/√((σ1²+σ2²)/2)
Fig5B # vesicles
Difference of means
mean = 23.8
0% < 0 < 100%
95% HDI
16.2 31.6
μ1 − μ2
Difference of SD
mode = 4.18
8% < 0 < 92%
95% HDI
-1.67 9.72
σ1 − σ2
Effect size
mode = 1.03
0% < 0 < 100%
95% HDI
0.686 1.42
(μ1−μ2)/√((σ1²+σ2²)/2)
Fig5E RRP
Difference of means
mean = 1480
2.6% < 0 < 97.4%
95% HDI
4.92 2990
μ1 − μ2
Difference of SD
mode = 976
1.4% < 0 < 98.6%
95% HDI
-10.4 2390
σ1 − σ2
Effect size
mode = 0.697
2.6% < 0 < 97.4%
95% HDI
-0.014 1.44
(μ1−μ2)/√((σ1²+σ2²)/2)
Fig5F resting pool
Difference of means
mean = 73.9
0.4% < 0 < 99.6%
95% HDI
21 127
μ1 − μ2
Difference of SD
mode = 14.9
17.1% < 0 < 82.9%
95% HDI
-20.4 63.8
σ1 − σ2
Effect size
mode = 0.984
0.4% < 0 < 99.6%
95% HDI
0.238 1.75
(μ1−μ2)/√((σ1²+σ2²)/2)

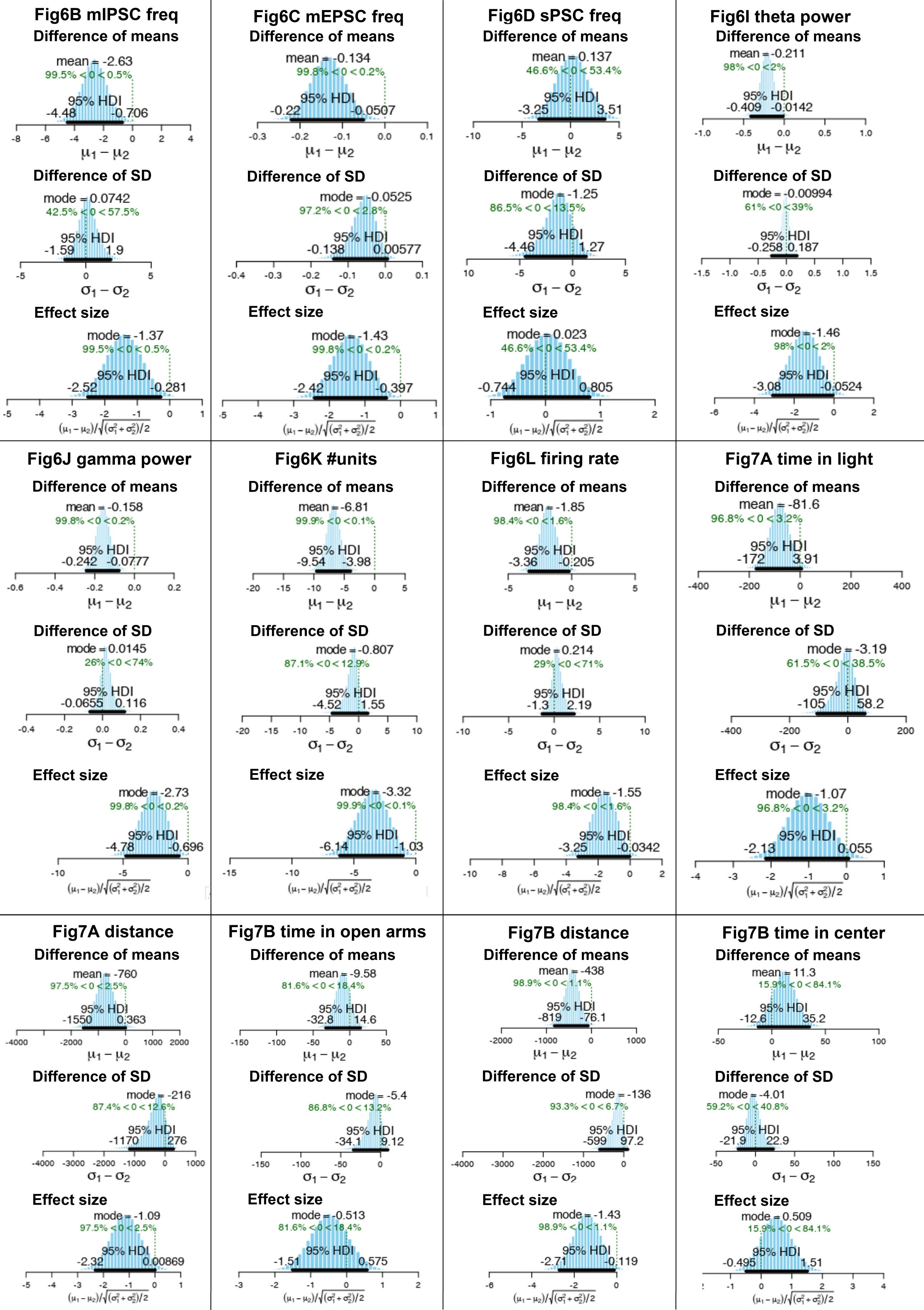
Fig6B mIPSC freq
Difference of means
mean = -2.63
99.5% < 0 < 0.5%
95% HDI
-4.48 -0.706
μ1 − μ2
Difference of SD
mode = 0.0742
42.5% < 0 < 57.5%
95% HDI
-1.59 1.9
σ1 − σ2
Effect size
mode = -1.37
99.5% < 0 < 0.5%
95% HDI
-2.52 -0.281
Fig6C mEPSC freq
Difference of means
mean = -0.134
99.8% < 0 < 0.2%
95% HDI
-0.22 -0.0507
Difference of SD
mode = -0.0525
97.2% < 0 < 2.8%
95% HDI
-0.138 0.00577
Effect size
mode = -1.43
99.8% < 0 < 0.2%
95% HDI
-2.42 -0.397
Fig6D sPSC freq
Difference of means
mean = 0.137
46.6% < 0 < 53.4%
95% HDI
-3.25 3.51
Difference of SD
mode = -1.25
86.5% < 0 < 13.5%
95% HDI
-4.46 1.27
Effect size
mode = 0.023
46.6% < 0 < 53.4%
95% HDI
-0.744 0.805
Fig6I theta power
Difference of means
mean = -0.211
98% < 0 < 2%
95% HDI
-0.409 -0.0142
Difference of SD
mode = -0.00994
61% < 0 < 39%
95% HDI
-0.258 0.187
Effect size
mode = -1.46
98% < 0 < 2%
95% HDI
-3.08 -0.0524
Fig6J gamma power
Difference of means
mean = -0.158
99.8% < 0 < 0.2%
95% HDI
-0.242 -0.0777
Difference of SD
mode = 0.0145
26% < 0 < 74%
95% HDI
-0.0655 0.116
Effect size
mode = -2.73
99.8% < 0 < 0.2%
95% HDI
-4.78 -0.696
Fig6K #units
Difference of means
mean = -6.81
99.9% < 0 < 0.1%
95% HDI
-9.54 -3.98
Difference of SD
mode = -0.807
87.1% < 0 < 12.9%
95% HDI
-4.52 1.55
Effect size
mode = -3.32
99.9% < 0 < 0.1%
95% HDI
-6.14 -1.03
Fig6L firing rate
Difference of means
mean = -1.85
98.4% < 0 < 1.6%
95% HDI
-3.36 -0.205
Difference of SD
mode = 0.214
29% < 0 < 71%
95% HDI
-1.3 2.19
Effect size
mode = -1.55
98.4% < 0 < 1.6%
95% HDI
-3.25 -0.0342
Fig7A time in light
Difference of means
mean = -81.6
96.8% < 0 < 3.2%
95% HDI
-172 3.91
Difference of SD
mode = -3.19
61.5% < 0 < 38.5%
95% HDI
-105 58.2
Effect size
mode = -1.07
96.8% < 0 < 3.2%
95% HDI
-2.13 0.055
Fig7A distance
Difference of means
mean = -760
97.5% < 0 < 2.5%
95% HDI
-1550 0.363
Difference of SD
mode = -216
87.4% < 0 < 12.6%
95% HDI
-1170 276
Effect size
mode = -1.09
97.5% < 0 < 2.5%
95% HDI
-2.32 0.00869
Fig7B time in open arms
Difference of means
mean = -9.58
81.6% < 0 < 18.4%
95% HDI
-32.8 14.6
Difference of SD
mode = -5.4
86.8% < 0 < 13.2%
95% HDI
-34.1 9.12
Effect size
mode = -0.513
81.6% < 0 < 18.4%
95% HDI
-1.51 0.575
Fig7B distance
Difference of means
mean = -438
98.9% < 0 < 1.1%
95% HDI
-819 -76.1
Difference of SD
mode = -136
93.3% < 0 < 6.7%
95% HDI
-599 97.2
Effect size
mode = -1.43
98.9% < 0 < 1.1%
95% HDI
-2.71 -0.119
Fig7B time in center
Difference of means
mean = 11.3
15.9% < 0 < 84.1%
95% HDI
-12.6 35.2
Difference of SD
mode = -4.01
59.2% < 0 < 40.8%
95% HDI
-21.9 22.9
Effect size
mode = 0.509
15.9% < 0 < 84.1%
95% HDI
-0.495 1.51

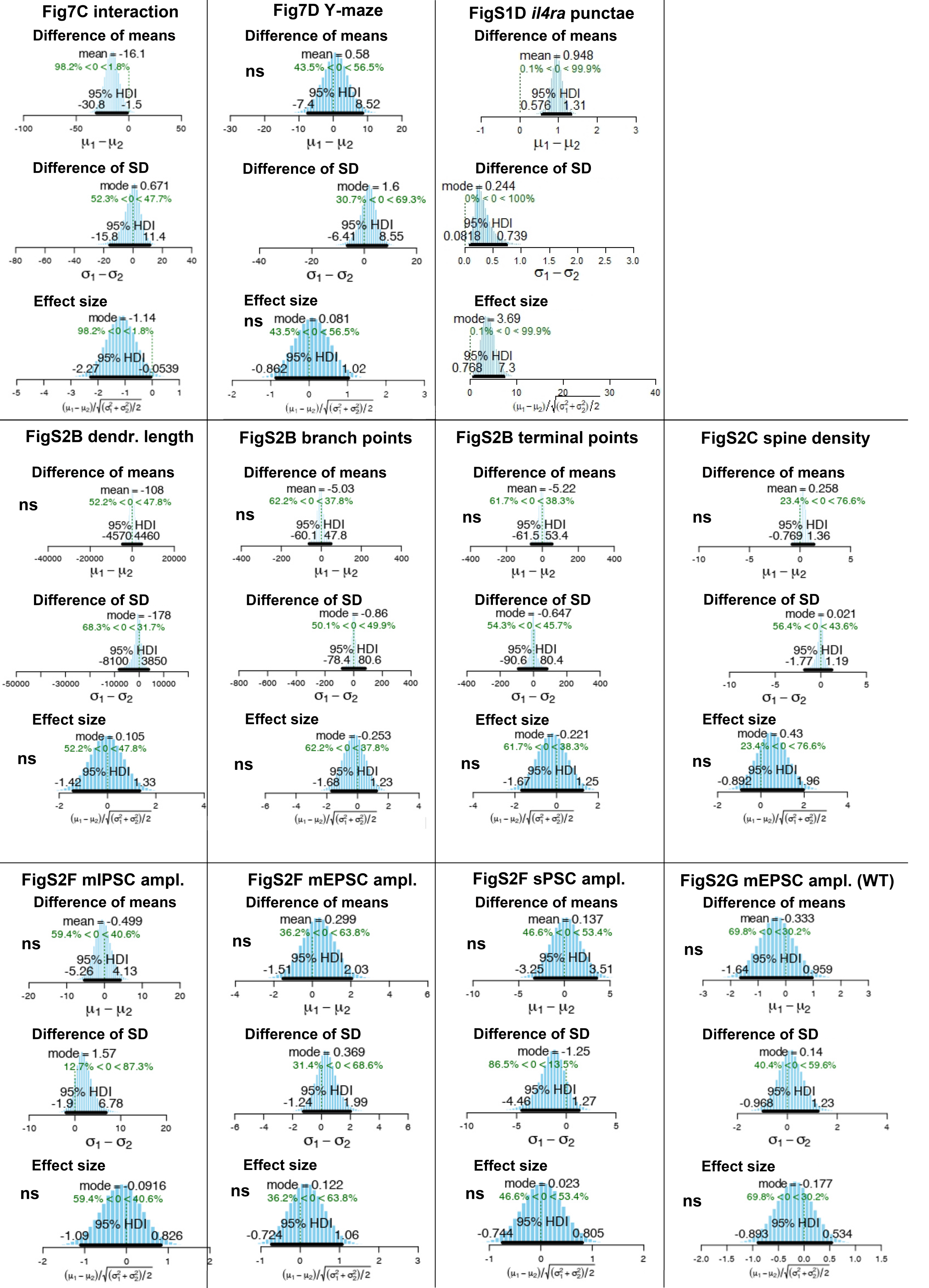

Fig7C interaction
Difference of means
mean = -16.1
98.2% < 0 < 1.8%
95% HDI
-30.8 -1.5
μ1 − μ2
Difference of SD
mode = 0.671
52.3% < 0 < 47.7%
95% HDI
-15.8 11.4
σ1 − σ2
Effect size
mode = -1.14
98.2% < 0 < 1.8%
95% HDI
-2.27 -0.0539
Fig7D Y-maze
Difference of means
ns
mean = 0.58
43.5% < 0 < 56.5%
95% HDI
-7.4 8.52
μ1 − μ2
Difference of SD
mode = 1.6
30.7% < 0 < 69.3%
95% HDI
-6.41 8.55
σ1 − σ2
Effect size
ns
mode = 0.081
43.5% < 0 < 56.5%
95% HDI
-0.862 1.02
FigS1D il4ra punctae
Difference of means
mean = 0.948
0.1% < 0 < 99.9%
95% HDI
0.576 1.31
μ1 − μ2
Difference of SD
mode = 0.244
0% < 0 < 100%
95% HDI
0.0818 0.739
σ1 − σ2
Effect size
mode = 3.69
0.1% < 0 < 99.9%
95% HDI
0.768 7.3
FigS2B dendr. length
Difference of means
ns
mean = -108
52.2% < 0 < 47.8%
95% HDI
-4570 4460
μ1 − μ2
Difference of SD
mode = -178
68.3% < 0 < 31.7%
95% HDI
-8100 3850
σ1 − σ2
Effect size
ns
mode = 0.105
52.2% < 0 < 47.8%
95% HDI
-1.42 1.33
FigS2B branch points
Difference of means
ns
mean = -5.03
62.2% < 0 < 37.8%
95% HDI
-60.1 47.8
μ1 − μ2
Difference of SD
mode = -0.86
50.1% < 0 < 49.9%
95% HDI
-78.4 80.6
σ1 − σ2
Effect size
ns
mode = -0.253
62.2% < 0 < 37.8%
95% HDI
-1.68 1.23
FigS2B terminal points
Difference of means
ns
mean = -5.22
61.7% < 0 < 38.3%
95% HDI
-61.5 53.4
μ1 − μ2
Difference of SD
mode = -0.647
54.3% < 0 < 45.7%
95% HDI
-90.6 80.4
σ1 − σ2
Effect size
ns
mode = -0.221
61.7% < 0 < 38.3%
95% HDI
-1.67 1.25
FigS2C spine density
Difference of means
ns
mean = 0.258
23.4% < 0 < 76.6%
95% HDI
-0.769 1.36
μ1 − μ2
Difference of SD
mode = 0.021
56.4% < 0 < 43.6%
95% HDI
-1.77 1.19
σ1 − σ2
Effect size
ns
mode = 0.43
23.4% < 0 < 76.6%
95% HDI
-0.892 1.96
FigS2F mIPSC ampl.
Difference of means
ns
mean = -0.499
59.4% < 0 < 40.6%
95% HDI
-5.26 4.13
μ1 − μ2
Difference of SD
mode = 1.57
12.7% < 0 < 87.3%
95% HDI
-1.9 6.78
σ1 − σ2
Effect size
ns
mode = -0.0916
59.4% < 0 < 40.6%
95% HDI
-1.09 0.826
FigS2F mEPSC ampl.
Difference of means
ns
mean = 0.299
36.2% < 0 < 63.8%
95% HDI
-1.51 2.03
μ1 − μ2
Difference of SD
mode = 0.369
31.4% < 0 < 68.6%
95% HDI
-1.24 1.99
σ1 − σ2
Effect size
ns
mode = 0.122
36.2% < 0 < 63.8%
95% HDI
-0.724 1.06
FigS2F sPSC ampl.
Difference of means
ns
mean = 0.137
46.6% < 0 < 53.4%
95% HDI
-3.25 3.51
μ1 − μ2
Difference of SD
mode = -1.25
86.5% < 0 < 13.5%
95% HDI
-4.46 1.27
σ1 − σ2
Effect size
ns
mode = 0.023
46.6% < 0 < 53.4%
95% HDI
-0.744 0.805
FigS2G mEPSC ampl. (WT)
Difference of means
ns
mean = -0.333
69.8% < 0 < 30.2%
95% HDI
-1.64 0.959
μ1 − μ2
Difference of SD
mode = 0.14
40.4% < 0 < 59.6%
95% HDI
-0.968 1.23
σ1 − σ2
Effect size
ns
mode = -0.177
69.8% < 0 < 30.2%
95% HDI
-0.893 0.534

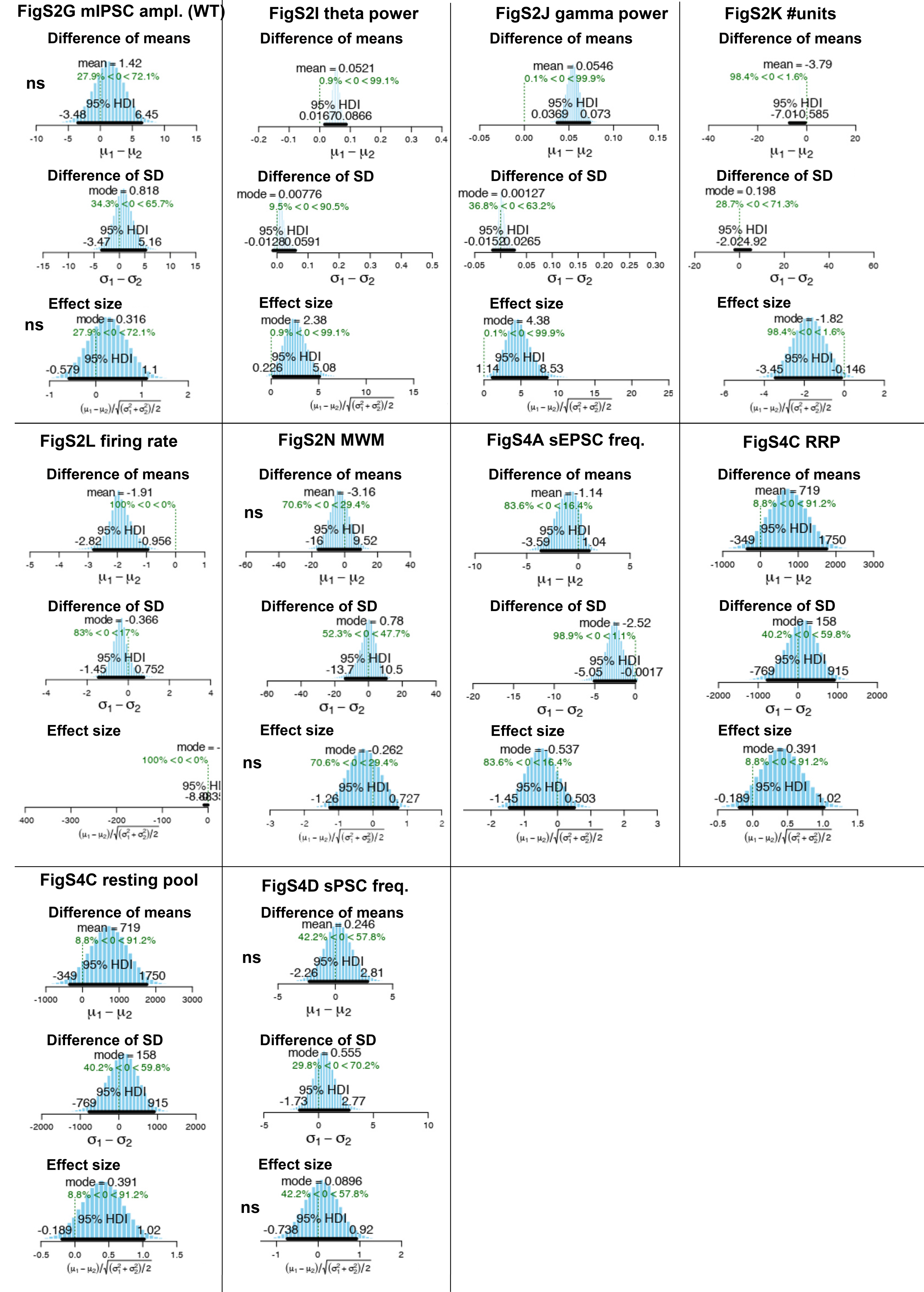

FigS2G mIPSC ampl. (WT)
Difference of means
ns
mean = 1.42
27.9% < 0 < 72.1%
95% HDI
-3.48
6.45
Difference of SD
mode = 0.818
34.3% < 0 < 65.7%
95% HDI
-3.47
5.16
Effect size
ns
mode = 0.316
27.9% < 0 < 72.1%
95% HDI
-0.579
1.1
FigS2I theta power
Difference of means
mean = 0.0521
0.9% < 0 < 99.1%
95% HDI
0.01670.0866
Difference of SD
mode = 0.00776
9.5% < 0 < 90.5%
95% HDI
-0.01280.0591
Effect size
mode = 2.38
0.9% < 0 < 99.1%
95% HDI
0.226
5.08
FigS2J gamma power
Difference of means
mean = 0.0546
0.1% < 0 < 99.9%
95% HDI
0.0369
0.073
Difference of SD
mode = 0.00127
36.8% < 0 < 63.2%
95% HDI
-0.01520.0265
Effect size
mode = 4.38
0.1% < 0 < 99.9%
95% HDI
1.14
8.53
FigS2K #units
Difference of means
mean = -3.79
98.4% < 0 < 1.6%
95% HDI
-7.010.585
Difference of SD
mode = 0.198
28.7% < 0 < 71.3%
95% HDI
-2.024.92
Effect size
mode = -1.82
98.4% < 0 < 1.6%
95% HDI
-3.45
-0.146
FigS2L firing rate
Difference of means
mean = -1.91
100% < 0 < 0%
95% HDI
-2.82
-0.956
Difference of SD
mode = -0.366
83% < 0 < 17%
95% HDI
-1.45
0.752
Effect size
100% < 0 < 0%
FigS2N MWM
Difference of means
ns
mean = -3.16
70.6% < 0 < 29.4%
95% HDI
-16
9.52
Difference of SD
mode = 0.78
52.3% < 0 < 47.7%
95% HDI
-13.7
10.5
Effect size
ns
mode = -0.262
70.6% < 0 < 29.4%
95% HDI
-1.26
0.727
FigS4A sEPSC freq.
Difference of means
mean = -1.14
83.6% < 0 < 16.4%
95% HDI
-3.59
1.04
Difference of SD
mode = -2.52
98.9% < 0 < 1.1%
95% HDI
-5.05
-0.0017
Effect size
mode = -0.537
83.6% < 0 < 16.4%
95% HDI
-1.45
0.503
FigS4C RRP
Difference of means
mean = 719
8.8% < 0 < 91.2%
95% HDI
-349
1750
Difference of SD
mode = 158
40.2% < 0 < 59.8%
95% HDI
-769
915
Effect size
mode = 0.391
8.8% < 0 < 91.2%
95% HDI
-0.189
1.02
FigS4C resting pool
Difference of means
mean = 719
8.8% < 0 < 91.2%
95% HDI
-349
1750
Difference of SD
mode = 158
40.2% < 0 < 59.8%
95% HDI
-769
915
Effect size
mode = 0.391
8.8% < 0 < 91.2%
95% HDI
-0.189
1.02
FigS4D sPSC freq.
Difference of means
ns
mean = 0.246
42.2% < 0 < 57.8%
95% HDI
-2.26
2.81
Difference of SD
mode = 0.555
29.8% < 0 < 70.2%
95% HDI
-1.73
2.77
Effect size
ns
mode = 0.0896
42.2% < 0 < 57.8%
95% HDI
-0.738
0.92
