# Supplementary figures and images for: Interleukin-4 receptor signaling modulates neuronal network activity
Source: J Exp Med. 2022 May 19;219(6):e20211887. doi: 10.1084/jem.20211887 (PMC9123307; doi:10.1084/jem.20211887)

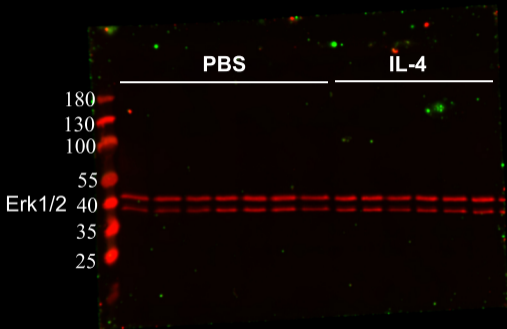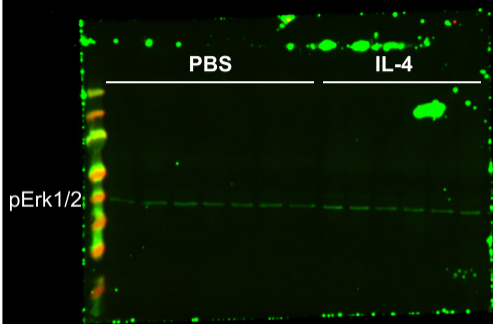

Supplement: SourceData F2 — contains original blots for Fig. 2. [file JEM_20211887_SourceDataF2.pdf]

Fig. 4L

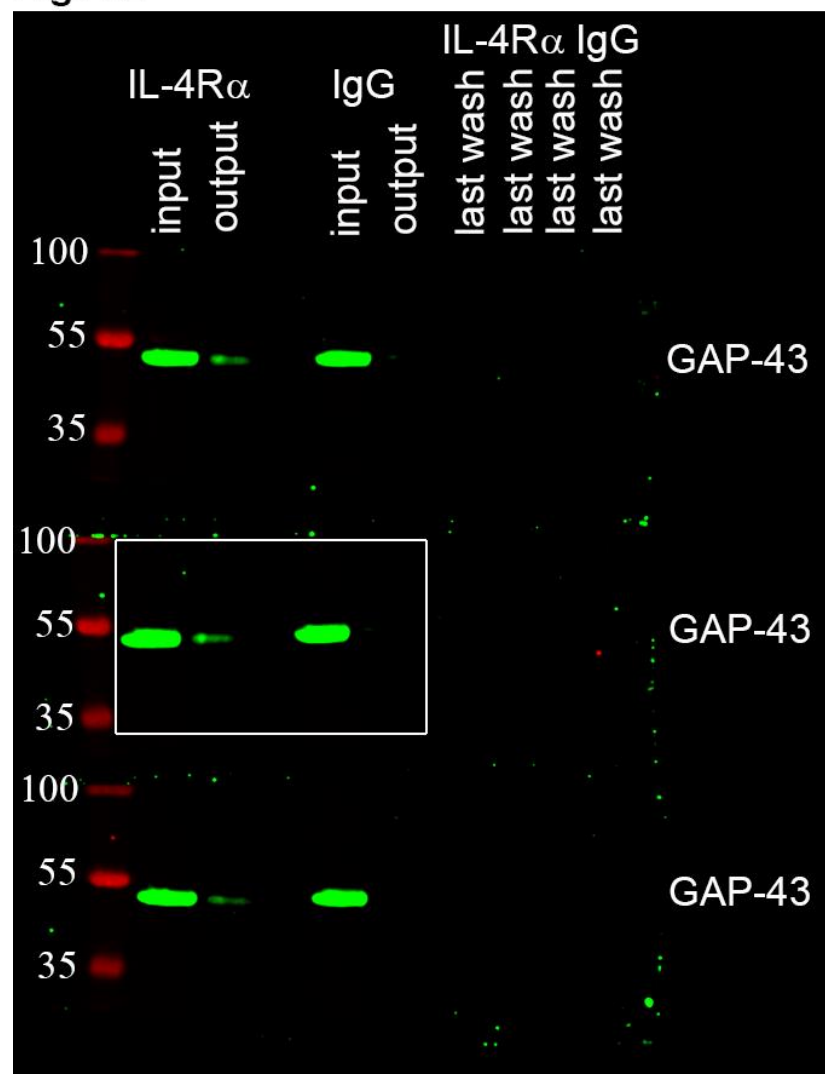

Fig. 4M

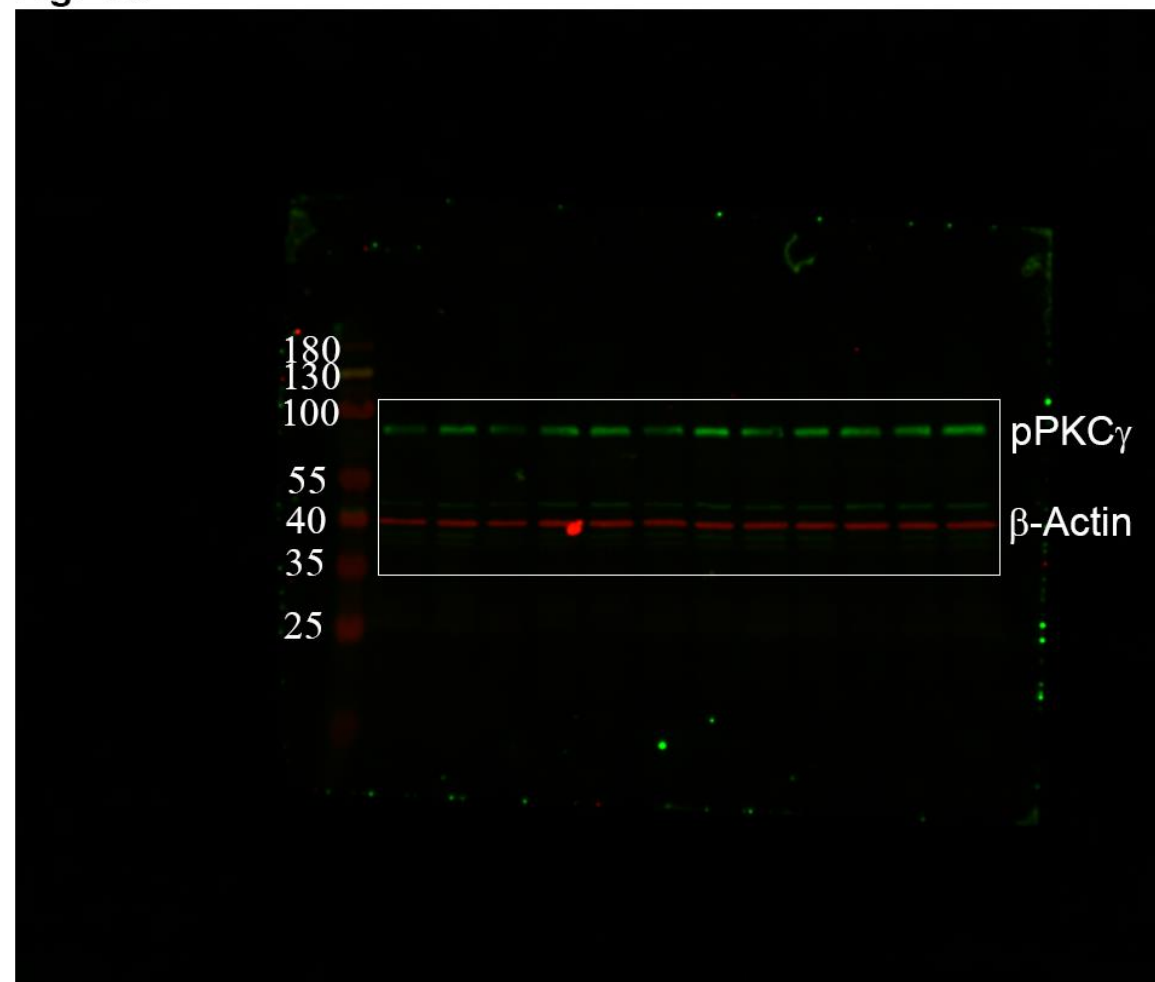

Supplement: SourceData F4 — contains original blots for for Fig. 4. [file JEM_20211887_SourceDataF4.pdf]
